# Liposomes embedded with PEGylated iron oxide nanoparticles enable ferroptosis and combination therapy in cancer

**DOI:** 10.1093/nsr/nwac167

**Published:** 2022-08-18

**Authors:** Yang Liu, Xuebo Quan, Jie Li, Jiawei Huo, Xing Li, Zhongpu Zhao, Shumu Li, Jing Wan, Jiao Li, Shuai Liu, Tao Wang, Xing Zhang, Bo Guan, Rui Wen, Zhenwen Zhao, Chunru Wang, Chunli Bai

**Affiliations:** Beijing National Research Center for Molecular Sciences, Key Laboratory of Molecular Nanostructure and Nanotechnology, Institute of Chemistry, Chinese Academy of Sciences, Beijing 100190, China; University of Chinese Academy of Sciences, Beijing 100049, China; Institute of Systems and Physical Biology, Shenzhen Bay Laboratory, Shenzhen 518107, China; Beijing National Research Center for Molecular Sciences, Key Laboratory of Molecular Nanostructure and Nanotechnology, Institute of Chemistry, Chinese Academy of Sciences, Beijing 100190, China; Beijing National Research Center for Molecular Sciences, Key Laboratory of Molecular Nanostructure and Nanotechnology, Institute of Chemistry, Chinese Academy of Sciences, Beijing 100190, China; University of Chinese Academy of Sciences, Beijing 100049, China; University of Chinese Academy of Sciences, Beijing 100049, China; Key Laboratory of Analytical Chemistry for Living Biosystems, Institute of Chemistry, Chinese Academy of Sciences, Beijing Mass Spectrum Center, Beijing 100190, China; Beijing National Research Center for Molecular Sciences, Key Laboratory of Molecular Nanostructure and Nanotechnology, Institute of Chemistry, Chinese Academy of Sciences, Beijing 100190, China; University of Chinese Academy of Sciences, Beijing 100049, China; Beijing National Research Center for Molecular Sciences, Key Laboratory of Molecular Nanostructure and Nanotechnology, Institute of Chemistry, Chinese Academy of Sciences, Beijing 100190, China; Beijing National Research Center for Molecular Sciences, Key Laboratory of Molecular Nanostructure and Nanotechnology, Institute of Chemistry, Chinese Academy of Sciences, Beijing 100190, China; University of Chinese Academy of Sciences, Beijing 100049, China; Beijing National Research Center for Molecular Sciences, Key Laboratory of Molecular Nanostructure and Nanotechnology, Institute of Chemistry, Chinese Academy of Sciences, Beijing 100190, China; Beijing National Research Center for Molecular Sciences, Key Laboratory of Molecular Nanostructure and Nanotechnology, Institute of Chemistry, Chinese Academy of Sciences, Beijing 100190, China; Beijing National Research Center for Molecular Sciences, Key Laboratory of Molecular Nanostructure and Nanotechnology, Institute of Chemistry, Chinese Academy of Sciences, Beijing 100190, China; Beijing National Research Center for Molecular Sciences, Key Laboratory of Molecular Nanostructure and Nanotechnology, Institute of Chemistry, Chinese Academy of Sciences, Beijing 100190, China; Beijing National Research Center for Molecular Sciences, Key Laboratory of Molecular Nanostructure and Nanotechnology, Institute of Chemistry, Chinese Academy of Sciences, Beijing 100190, China; Beijing National Research Center for Molecular Sciences, Key Laboratory of Molecular Nanostructure and Nanotechnology, Institute of Chemistry, Chinese Academy of Sciences, Beijing 100190, China; University of Chinese Academy of Sciences, Beijing 100049, China; Key Laboratory of Analytical Chemistry for Living Biosystems, Institute of Chemistry, Chinese Academy of Sciences, Beijing Mass Spectrum Center, Beijing 100190, China; Beijing National Research Center for Molecular Sciences, Key Laboratory of Molecular Nanostructure and Nanotechnology, Institute of Chemistry, Chinese Academy of Sciences, Beijing 100190, China; University of Chinese Academy of Sciences, Beijing 100049, China; Beijing National Research Center for Molecular Sciences, Key Laboratory of Molecular Nanostructure and Nanotechnology, Institute of Chemistry, Chinese Academy of Sciences, Beijing 100190, China; University of Chinese Academy of Sciences, Beijing 100049, China

**Keywords:** liposome, ultrasmall iron oxide nanoparticles, intrabilayer lipid peroxidation, ferroptosis, combination therapy

## Abstract

Ferroptosis, an iron-dependent regulated cell death process driven by excessive lipid peroxides, can enhance cancer vulnerability to chemotherapy, targeted therapy and immunotherapy. As an essential upstream process for ferroptosis activation, lipid peroxidation of biological membranes is expected to be primarily induced by intrabilayer reactive oxygen species (ROS), indicating a promising strategy to initiate peroxidation by improving the local content of diffusion-limited ROS in the lipid bilayer. Herein, liposomes embedded with PEG-coated 3 nm γ-Fe_2_O_3_ nanoparticles in the bilayer (abbreviated as Lp-IO) were constructed to promote the intrabilayer generation of hydroxyl radicals (•OH) from hydrogen peroxide (H_2_O_2_), and the integration of amphiphilic PEG moieties with liposomal bilayer improved lipid membrane permeability to H_2_O_2_ and •OH, resulting in efficient initiation of lipid peroxidation and thus ferroptosis in cancer cells. Additionally, Lp-IO enabled traceable magnetic resonance imaging and pH/ROS dual-responsive drug delivery. Synergistic antineoplastic effects of chemotherapy and ferroptosis, and alleviated chemotherapeutic toxicity, were achieved by delivering doxorubicin (capable of xCT and glutathione peroxidase inhibition) with Lp-IO. This work provides an efficient alternative for triggering therapeutic lipid peroxidation and a ferroptosis-activating drug delivery vehicle for combination cancer therapies.

## Teaser

Liposomes embedded with PEGylated ultrasmall iron oxide nanoparticles integrate ferroptosis activation, MRI tracing, and pH/ROS-sensitive drug delivery for combination cancer therapies.

## INTRODUCTION

In clinical practice, poor efficacy and undesirable side effects of chemotherapy remain essentially insurmountable challenges. Combination treatment with existing drugs is already a cornerstone of cancer therapy. Ferroptosis is a new non-apoptotic programmed cell death resulting from the iron-dependent accumulation of lipid peroxides (LPOs) to lethal levels [1]. Cancer cells have a higher demand for iron accumulation, fatty acid synthesis, activated autophagic flux and epithelial-mesenchymal transition (EMT) to maintain malignant proliferation. These hallmarks enhance the susceptibility of various cancer types to ferroptosis [2]. Inducing ferroptosis is a sound therapeutic strategy to abolish the resistance to conventional cytotoxic and targeted agents by targeting the glutathione (GSH) and/or glutathione peroxidase (GPX-4) dependency of diverse therapy-resistant cancer cells [3]. Furthermore, chemotherapy and targeted therapy enhance ferroptosis sensitivity via reactive oxygen species (ROS) accumulation, iron enrichment, GSH depletion and GPX-4 inactivation [4].

Ferroptosis-dependent lipid peroxidation is a radical-mediated chain reaction involving initiation, propagation and termination processes [5]. Hydroxyl radicals (•OH), transition metal ions (e.g. iron redox couples) and lipoxygenase trigger the initiation phase, which involves hydrogen atom abstraction at the bis-allylic site (C−H bond with a C=C double bond on either side) of the lipid, with a polyunsaturated fatty acid (PUFA) moiety to generate an alkyl radical. The unstable alkyl radical readily combines with oxygen (O_2_) to yield a peroxyl radical, propagating peroxidation by abstracting a hydrogen atom from another neighboring unsaturated lipid (UL) to produce excess LPOs. For free PUFAs in solution, H-atom transfer from PUFAs to peroxyl radicals in the propagation phase is generally considered the rate-limiting step of peroxidation [6]. Notably, the peroxidation on membrane ULs containing PUFAs, but not free PUFAs, is responsible for cell ferroptosis [2,7]. Bis-allylic hydrogen atoms of ULs in biological membranes, as the leading reaction sites for peroxidation initiation and propagation, are primarily located in the hydrophobic interior of the lipid bilayer [7,8]. The hydrophobic region acts as a significant thermodynamic barrier for hydrophilic polar non-electrolytes (e.g. hydrogen peroxide (H_2_O_2_), •OH and •OOH) and ions to diffuse toward the center of the bilayer, while O_2_, with slight hydrophobicity, can freely diffuse across membranes [9,10]. For lipid peroxidation in membranes, O_2_, which has high membrane permeability, can be adequately supplied to propagate peroxidation, and thus, the initiation reaction induced by diffusion-restricted •OH/H_2_O_2_ is expected to become the predominant process. Molecular dynamics (MD) simulations suggest that •OH has a lower permeation energy barrier than H_2_O_2_ [11], but the extremely high reactivity and thus ultrashort lifetime (∼1 ns) abrogates its diffusion across membranes [6,12,13]. As •OH reacts with almost any neighboring components at diffusion-controlled rates, the peroxidation initiation by •OH should be dominated by its local content in the lipid bilayer. Therefore, increasing the intrabilayer yield of •OH could be an essential strategy to trigger lipid peroxidation for ferroptosis activation.

Iron-based nanomaterials (e.g. amorphous iron [14], Fe_2_O_3_ [15] and Fe_3_O_4_ [16]) converting H_2_O_2_ to •OH via the Fenton reaction are commonly utilized as ferroptosis activators, but high doses of Fe (e.g. 10 mg/kg for ferumoxytol and 75 mg/kg for amorphous iron nanoparticles) are required for ferroptosis-based cancer therapy [17]. It is difficult for iron-based nanoparticles (NPs) to become enriched in the lipid bilayer after cell uptake and to produce •OH there, and the •OH generated in the cytoplasm is prevented from initiating intrabilayer lipid peroxidation, probably resulting in limited ferroptosis activation by iron-based nanomaterials. In addition, as even overexpressed H_2_O_2_ in cancer cells remains at a relatively low level, ferroptosis could be enhanced by introducing exogenous/endogenous H_2_O_2_ or a highly reactive iron redox couple (Fe^2+^−Fe^3+^) [18–20]. As such, we believe that embedding iron-based NPs in the lipid bilayer would be a viable approach to enable the intrabilayer generation of sufficient •OH to initiate lipid peroxidation and further induce ferroptosis with high efficiency. Liposomes, popular drug delivery vehicles for improving pharmacokinetics and alleviating side effects, are spherical vesicles with a lipophilic bilayer sandwiched structure and can be manipulated to consist of large amounts of ULs [21]. Amphiphilic poly(ethylene glycol) (PEG) was found to enhance the permeability of liposomal membranes [22,23]. Both γ-Fe_2_O_3_ and Fe_3_O_4_ NPs exhibit peroxidase-like activities [24] and are available as magnetic resonance imaging (MRI) contrast agents for their intrinsic superparamagnetism [15,16]. As Fe_3_O_4_ NPs could release Fe^2+^ and Fe^3+^ ions to form the iron redox couple for peroxidation initiation in the absence of H_2_O_2_, embedding Fe_3_O_4_ NPs within liposomal membranes may interplay with the intrabilayer peroxidation and facilitate the autoxidation of liposomes, causing stability issues. In addition, compared with Fe_3_O_4_ NPs, γ-Fe_2_O_3_ NPs possess higher biosafety [25]. Ultrasmall γ-Fe_2_O_3_ NPs (∼3 nm) are chosen to be inserted into the bilayer (thickness ranging from 3.4 nm to 4.4 nm) of liposomes [26].

Consequently, liposomes embedded with PEG-coated ultrasmall γ-Fe_2_O_3_ NPs in the lipid bilayer were prepared (abbreviated as Lp-IO). Lp-IO promoted the intrabilayer yield of •OH from diffusion-limited H_2_O_2_ via the Fenton reaction and enhanced the permeability of liposomal membranes to H_2_O_2_ and •OH, as revealed by MD simulations. Lp-IO enabled the efficient initiation of lipid peroxidation and thus triggered ferroptosis for cancer therapy *in vitro* and *in vivo*. In addition, Lp-IO, as a drug delivery vehicle, is traceable by MRI and capable of pH/ROS-responsive release. Chemotherapeutic doxorubicin (DOX) delivered by Lp-IO had a synergistic antitumor effect of ferroptosis and chemotherapy, and significantly reduced toxicity. We provide an efficient strategy to activate ferroptosis for combination cancer therapies.

## RESULTS AND DISCUSSION

### Preparation, characterization, lipid peroxidation and MD simulation of Lp-IO

**Figure 1.**
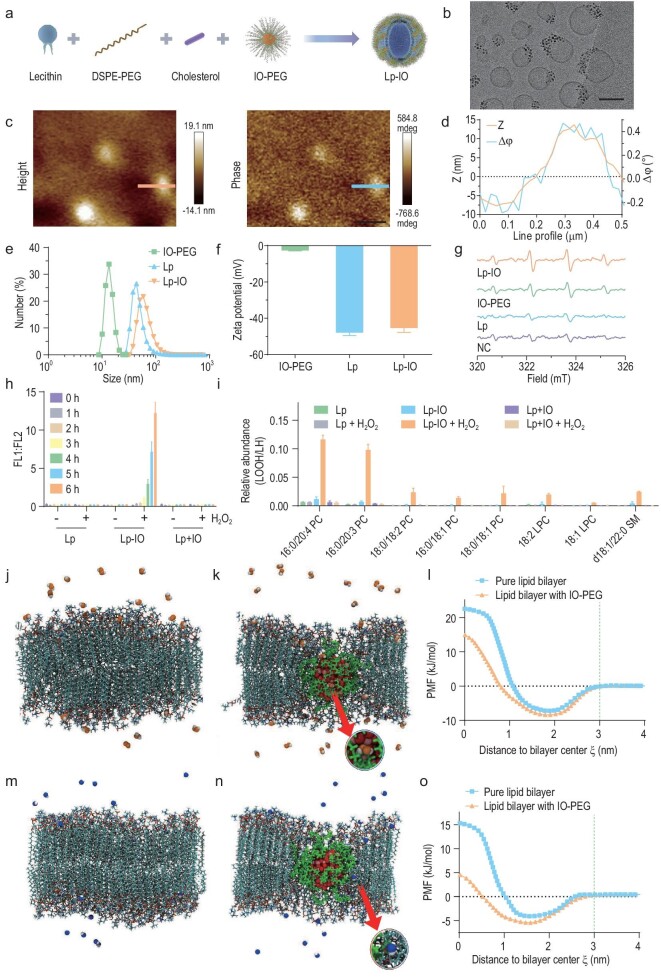
Preparation, characterization, lipid peroxidation and MD simulations of Lp-IO. (a) Schematic preparation process. (b) Cyro-TEM image of Lp-IO; scale bar: 50 nm. (c) AFM (left) and MFM (right) images of Lp-IO; scale bars: 400 nm. (d) Profile analysis of line in Fig. 1c. (e) Hydrodynamic size distribution and (f) zeta potential of IO-PEG, Lp and Lp-IO. (g) X-band EPR spectra of DMPO captured hydroxyl radicals produced by H_2_O_2_ in the presence of ultrapure water (NC), Lp, IO-PEG and Lp-IO. (h and i) Lp, Lp-IO and Lp + IO were separately treated in the presence or absence of 1 mM H_2_O_2_. (h) The relative ratio of FL1 (green FL) to FL2 (red FL) from C11-BODIPY. (i) The peak area ratio of lipid hydroperoxide (LOOH) of PC, LPC and SM to12 : 0 LPC (internal standard) after treatment for 6 h. LOQ, the limit of quantification. (j and k) Typical equilibrated snapshots of H_2_O_2_ interacting with the pure lipid bilayer and the IO-PEG-doped lipid bilayer. The phospholipids (hydrogen, white; oxygen, red; nitrogen, dark blue; carbon, cyan; phosphorus, tan) and PEG chains (green) are displayed in licorice mode; the IONP (oxygen, red; iron, pink) and H_2_O_2_ molecules (hydrogen, white; oxygen, orange) are represented as van der Walls (vdW) spheres. Water molecules are not shown for clarity. (l) PMF profiles for a single H_2_O_2_ to penetrate the lipid bilayers. (m and n) Typical equilibrated snapshots of the interaction between •OH and lipid bilayers; the •OH is represented as vdW spheres (hydrogen, white; oxygen, blue). (o) PMF profiles for a single •OH to penetrate lipid bilayers.

Ultrasmall iron oxide nanoparticles (IONPs) with diameters of 2.2 nm and 3 nm, phosphorylated polyethylene glycol (PEG-PO), and PEG-PO-coated IONPs (IO-PEG) were prepared as reported [15]. PEG-PO was characterized by ^1^H, ^13^C and ^31^P nuclear magnetic resonance spectroscopy (Fig. S1 in the online supplementary file). Pristine liposomes (Lp) and Lp-IO were prepared by sonication (Fig. 1a). According to the magnetic hysteresis loop, 3 nm IONPs achieved a higher saturation magnetization than 2.2 nm IONPs (Fig. S2), indicating their better MRI enhancement capability and superiority for further investigations. Transmission electron microscopy (TEM) images and X-ray diffraction (XRD) patterns revealed the maghemite (γ-Fe_2_O_3_) crystal structure of IONPs (Fig. S3a–c). As revealed by X-ray photoelectron spectroscopy (XPS) analysis, IONPs with Fe 2p_3/2_ (711.4 eV), 2p_1/2_ (724.6 eV) and 3p (56.2 eV) peaks are composed of Fe^3+^ ions (Fig. S3d–g). IONPs were modified with PEG-PO to obtain amphiphilic IO-PEG, as confirmed by the coexistence of Fe 2p, Fe 3p and P 2p XPS peaks (Fig. S4a and b). IO-PEG maintains a uniform size distribution of ∼3 nm and a maghemite structure (Fig. S5). IO-PEG NPs were successfully inserted into the liposome bilayer and presented an aggregated morphology (Fig. 1b), probably resulting from the interchain entanglement between amphiphilic PEG and lipid alkane chains. The structure of Lp-IO was further verified by the coincidence of height (Z) and magnetic (Δϕ, phase) signals via atom force microscopy (AFM) and magnetic force microscopy (MFM) (Fig. 1c and d). In contrast, IO-PEG directly mixed with Lp (Lp + IO) gave magnetic signals only distributed around the Lp edge, and free Lp had a negligible magnetic response (Fig. S6a and b). In addition, Lp-IO gave rise to the characteristic XPS peaks of Fe^3+^ and the same XRD pattern of γ-Fe_2_O_3_ crystal as IO-PEG (Figs S4a and c, and S7). The encapsulation efficiency (EE) and the loading efficiency (LE) of Lp for IO-PEG were evaluated by the Fe content of Lp-IO via inductively coupled plasma mass spectrometry (ICP–MS) (Fig. S8). The EE reached a maximum value (70.3%) when the feed ratio reached 8.1 mg IO-PEG (containing 3 mg Fe) per 40 mg lecithin (a major ingredient of Lp), and the corresponding LE was 13.8%. The average hydrodynamic size of IO-PEG increases to ∼10 nm with a polydispersity index (PDI) of 0.14, which is attributed to the hydration effect of the modified PEG moieties. Compared with Lp (average: 51.90 ± 14.03 nm, PDI: 0.12 ± 0.04), Lp-IO (average: 61.9 ± 5.37 nm, PDI: 0.19 ± 0.01) has almost the same size distribution and dispersibility (Figs 1e and S9). The zeta potential of IO-PEG (−2.67 ± 0.22 mV) is quite low, and those of Lp (−48.00 ± 1.56 mV) and Lp-IO (−45.40 ± 2.42 mV) are similar (Fig. 1f), revealing that Lp-IO maintains the physicochemical nature and stability of Lp. Lp and Lp-IO have good stability in the physiological environment (Fig. S10). IO-PEG is stable in serum but not in PBS; this is probably attributed to protein corona formation, which improves its stability in serum. In addition, the surface PEG moieties of Lp-IO can alleviate rapid renal and immune clearance, promoting biocompatibility and long-term circulation in blood. Therefore, liposomes with ultrasmall IO-PEG embedded in the lipid bilayer were prepared and characterized as designed.

Iron oxide NPs exhibit peroxidase-mimetic properties, resulting from the NP surface as a heterogeneous Fenton system and the release of free Fe ions with catalytic activity [27]. Through the Fenton reaction, IO-PEG and Lp-IO both converted H_2_O_2_ to •OH radicals, which were trapped by 5,5-dimethyl-1-pyrroline N-oxide (DMPO) and detected via electron spin resonance (ESR) (Fig. 1g). The generated •OH could react extremely rapidly with adjacent ULs, triggering lipid peroxidation and LPO accumulation. The relative content of LPOs was quantified by the intensity ratio of green and red fluorescence (FL1 : FL2) from a fluorescent probe (C11-BODIPY). In the presence of H_2_O_2_, Lp-IO produced LPOs with time dependence (Fig. 1h), and either Lp or Lp + IO induced negligible LPOs. These results support the hypothesis that embedding IO-PEG within liposome membranes enables high-efficiency lipid peroxidation. The composition of oxygenated lipid species is a critical determinant of ferroptosis sensitivity [7,28]. Consequently, the major classes of lipids, including phosphatidylcholines (PC), phosphatidylethanolamine (PE), phosphatidylinositol (PI), sphingomyelin (SM) and lysophosphatidylcholine (LPC), were identified and quantified via liquid chromatography-mass spectrometry (LC-MS), revealing almost the same lipid composition for Lp and Lp-IO (Figs S11 and S12, and Table S1). The ULs with a >2.5-fold increase in the relative peak area ratio of doubly oxygenated lipid (LOOH) to 12 : 0 LPC (internal standard) in the presence versus the absence of H_2_O_2_ were considered be significantly peroxidized. Unsaturated PC, LPC and SM were oxygenated in Lp-IO, and the peroxidation vulnerability of PC/LPC/SM was generally enhanced with their degree of unsaturation (Figs 1i and S13). Upon initiation of lipid peroxidation, the length, number and position of double bonds in the ULs become dominant factors in the rate-limiting propagation step [6,29]. PE containing arachidonic acid (AA) and adrenoyl (AdA) in endoplasmic reticulum-associated compartments has been identified as the crucial phospholipid for GPX4-deficient ferroptosis [30]. However, unsaturated PEs with a comparably high content in Lp-IO, including 16 : 0/20 : 4 PE, 18 : 0/18 : 2 PE, 16 : 0/18 : 2 PE and 16 : 0/18 : 1 PE, did not undergo peroxidation as unsaturated PC/LPC did in the presence of H_2_O_2_. It has been revealed that NPs can alter membrane curvature, thus inducing the accumulation of specific lipids and lipid exchange in the curvature region [31]. IO-PEG NPs trigger the selective peroxidation on PC/LPC/SM (Fig. 1i), indicating that IO-PEG NPs probably interact with PC/LPC/SM and thus alter the distribution of both IO-PEG NPs and these lipids in liposomes. For Lp and Lp + IO, the peroxidation of most ULs, including PC/LPC, was not improved by H_2_O_2_, corresponding with the negligible LPO yield revealed by the C11-BODIPY probe (Fig. 1h). The selective peroxidation of PC/LPC/SM in Lp-IO indicates a potential strategy to amplify intrabilayer lipid peroxidation by improving PC/LPC/SM content in liposomes. Therefore, it was verified that embedding iron oxide NPs into the liposome bilayer efficiently initiated lipid peroxidation and generated excessive LPOs, probably activating ferroptosis *in vitro* and *in vivo*.

The initiation of lipid peroxidation in Lp-IO is dependent on the intrabilayer yield of •OH from H_2_O_2_, but the intralayer diffusion of H_2_O_2_ is usually limited by the hydrogen bonds formed between H_2_O_2_ molecules and the polar lipid headgroups. As PEG in the lipid bilayer is expected to improve the penetration of polar ROS, MD simulations were conducted to evaluate the permeability of liposome membranes to H_2_O_2_ and •OH in the presence or absence of embedded IO-PEG NPs. Simplified theoretical models of IO-PEG, pure lipid bilayer and IO-PEG-embedded lipid bilayer were constructed for the MD simulation (Fig. S14). The interactions between H_2_O_2_ and lipid bilayers were first simulated (Fig. S15). H_2_O_2_ only contacted the lipid head regions and did not penetrate the lipid bilayer during the simulation (Figs 1j and S16a, and Movie S1). For the IO-PEG-doped lipid bilayer, H_2_O_2_ traversed along the junction of the lipids and IO-PEG (Figs 1k and S16b, and Movie S2). Furthermore, the potential of mean force (PMF) for the translocation of a single H_2_O_2_ across the lipid bilayer was calculated (Fig. 1l). The energy barrier for H_2_O_2_ to penetrate the lipid bilayer was 29.74 kJ/mol, which decreased to ∼23.31 kJ/mol after inserting IO-PEG into the bilayer. This result verifies that the surface PEG moieties of IO-PEG could integrate with bilayer lipids to enhance the permeation to H_2_O_2_. In addition, the interactions between •OH and lipid bilayers were also investigated (Fig. S17). Consistent with H_2_O_2_, •OH did not enter the pure lipid bilayer (Figs 1m and S18a, and Movie S3), thus limiting the intrabilayer diffusion of free •OH to initiate lipid peroxidation in biological membranes. The embedding of IO-PEG in the lipid bilayer also promoted the permeability of •OH (Figs 1n and S18b, and Movie S4), and the permeation barrier decreased from 19.39 to 9.98 kJ/mol (Fig. 1o). H_2_O_2_ has a higher permeation energy barrier than •OH as a result of forming more hydrogen bonds with lipid head regions [32]. To further evaluate how fast H_2_O_2_ and •OH diffuse through the lipid bilayers, the permeability coefficient (P_m_) was calculated from the PMF. The P_m_ values of H_2_O_2_ and •OH to cross the pure lipid bilayer were 0.00315 and 0.152 cm s^−1^, respectively, and those for the lipid bilayer inserted with IO-PEG increased to 0.0476 and 2.69 cm s^−1^, respectively (Fig. S19). The calculated P_m_ for H_2_O_2_ to traverse the pure lipid bilayer is consistent with the reported experimental value [10], indicating that the MD simulations are credible. Therefore, the anchoring of IO-PEG in the lipid bilayer can facilitate the intrabilayer diffusion of H_2_O_2_ and •OH by lowering the free energy barrier of membrane permeation, thus promoting the intrabilayer yield of •OH and the interaction between •OH and UL to produce lethal LPOs.

### Ferroptosis activation by Lp-IO *in vitro*

**Figure 2.**
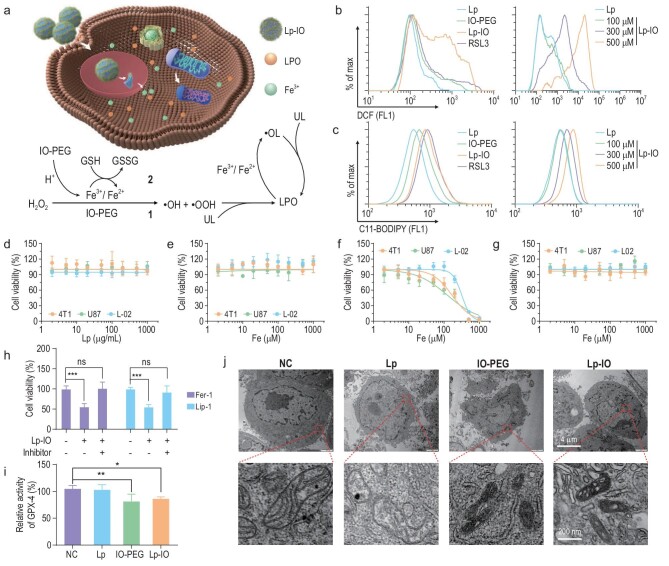
Lp-IO induces ferroptosis in cancer cells. (a) Schematic mechanism of the ferroptosis activation. (b) ROS and (c) LPO levels in 4T1 cells treated with (left) Lp, IO-PEG, Lp-IO and RSL3, and (right) Lp and Lp-IO at gradient concentrations. (d–g) Viability of 4T1, U87 and L-02 cells treated with (d) Lp, (e) IO-PEG, (f) Lp-IO and (g) UL-free Lp-IO. (h) Viability of 4T1 cells under Lp-IO treatment for 24 h in the presence and absence of ferroptosis inhibitor (Fer-1 or Lip-1). (i) Relative GPX-4 level and (j) TEM imaging of 4T1 cells treated with Lp, IO-PEG and Lp-IO. Mean ± s.d.; *n* = 6; *P < 0.05, **P < 0.01 and ***P < 0.001.

Lp-IO is expected to dissociate and release Fe^3+^ ions in acidic environments. The IO-PEG and Fe^3+^ ions will commonly convert H_2_O_2_ to •OH and •OOH via the Fenton reaction, quickly oxidizing the nearby ULs in the Lp-IO bilayer to LPOs. A portion of the released Fe^3+^ ions could be reduced to Fe^2+^ ions by cytoplastic GSH, catalyzing intracellular ULs to lipid peroxides and thus enhancing ferroptosis (Fig. 2a). For comparison, UL-free Lp-IO was prepared by embedding IO-PEG NPs in the bilayer of liposomes consisting of 1,2-dipalmitoyl-sn-glycero-3-phosphocholine (DPPC, 16 : 0 PC). Compared with Lp-IO, UL-free Lp-IO gets a dispersion of IO-PEG NPs within liposomal membranes, a lower yield, a larger hydrodynamic size and reduced stability (Fig. S20a–e), indicating that the composition of multiple lipids may be necessary for the construction of Lp-IO. In 4T1 cells (breast cancer cell line), Lp-IO induces ROS generation with time and dose dependence as revealed by a 2′,7′-dichlorofluorescin diacetate (DCF-DA) assay (Figs 2b and S21a). A GPX-4 inhibitor (RSL3) causing ROS accumulation was utilized to facilitate the increase of DCF fluorescence (FL) as a positive control. Subsequently, Lp-IO improved LPO levels *in vitro* with time and dose dependence, as evaluated by the green FL of C11-BODIPY (Figs 2c and S22a). Lp-IO also triggered ROSs and LPOs in A549 cells (Figs S21b, S22b and S23). Intracellular ROSs and LPOs generated by UL-free Lp-IO were negligible (Fig. S20f and g), indicating the critical role of unsaturated lipids for Lp-IO.

Cancer cells always have high levels of H_2_O_2_ in the cytoplasm and tumor microenvironment (TME), which is attributed to increased H_2_O_2_ production and impaired redox homeostasis [33]. Neither Lp nor IO-PEG induced apparent toxicity in cancerous (4T1 and U87 (human glioblastoma cell line)) and non-cancerous (L-02, human normal liver cell line) cells at maximum doses (1000 μg/mL for Lp; 1000 μM Fe for IO-PEG) (Fig. 2d and e). Lp-IO was primarily internalized via caveolin-mediated endocytosis (Fig. S24) and showed significant cell inhibition with half-maximal inhibitory concentrations (IC_50_) of 168.5, 125.3 and 318.0 μM in 4T1, U87 and L-02 cells, respectively (Fig. 2f). The enhanced inhibition in cancer cells was ascribed to their comparably high levels of H_2_O_2_. In contrast, UL-free Lp-IO and the mixture of Lp and IO-PEG exhibited negligible cytotoxicity (Figs 2g and S25). In addition, although the cell culture medium contained various free PUFAs, IO-PEG could not induce cell inhibition in the medium. Ferrostatin-1 (Fer-1) and liproxstatin-1 (Lip-1), as ferroptosis inhibitors, alleviated the inhibitory effect of Lp-IO (Fig. 2h). GPX-4 is a phospholipid hydroperoxidase that protects cells from lipid peroxidation damage, and the degradation of GPX-4 is a downstream marker of ferroptosis. Lp-IO reduced the activity and expression of GPX-4 in 4T1 cells (Figs 2i and S26). In addition, other hallmarks of ferroptosis were observed after Lp-IO treatment, including shrunken morphology, dense membranes and reduced cristae (Fig. 2j). Therefore, Lp-IO enables lipid peroxidation *in vitro* and further triggers the ferroptosis of cancer cells. Notably, IO-PEG reduced GPX-4 ability and produced shrunken and dense mitochondria but did not induce distinct cytotoxicity. This is probably attributed to the yield of •OH in the cytoplasm by IO-PEG, which could attack mitochondria but could not effectively induce ferroptosis.

### MRI and the antineoplastic effect of Lp-IO *in vivo*

Superparamagnetic IONPs are common T_2_-weighted MRI contrast agents, indicating that Lp-IO can be traced by MRI. According to the magnetic hysteresis loops (Fig. S27), IONPs, IO-PEG and Lp-IO are superparamagnetic. Their saturation magnetization (M_s_) values based on Fe content were found to be 45.4, 43.7 and 44.4 emu/g, respectively. Similar M_s_ values indicated that the magnetic nature of IONPs was maintained after modification with PEG-PO and subsequent doping in liposomes. Relaxivity is the extent to which a contrast agent can enhance the relaxation rate of tissue water, and longitudinal and transverse relaxivities are denoted r_1_ and r_2_, respectively. Under a high magnetic field (7.0 T) for *in vivo* MRI, the r_1_ relaxivities of IO-PEG and Lp-IO are 0.71 and 0.21 mM^−1^s^−1^, and the r_2_ values are 30.7 and 62.7 mM^−1^s^−1^, respectively (Fig. S28). The r_2_ of Lp-IO is twice as high as IO-PEG, attributing to the magnetic dipole interaction (MDI) of the aggregated IO-PEG NPs in the bilayer of Lp-IO [34]. Besides, water penetration is hindered by the clustering of IO-PEG in Lp-IO, resulting in low r_1_ relaxivity. The high r_2_/r_1_ ratio (297) of Lp-IO makes it preferable for T_2_ contrast enhancement. Lp-IO exhibited a much better T_2_-weighted MRI enhancement than IO-PEG in a concentration-dependence at 7.0 T (Fig. S29).

**Figure 3.**
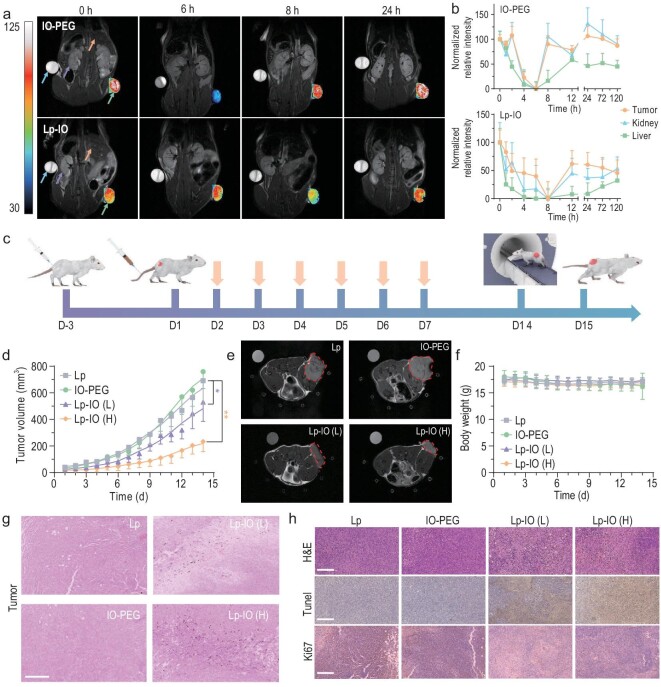
MRI and antitumor behavior of Lp-IO. (a) Pseudo-color T_2_-weighted MR images of 4T1 tumor-bearing Balb/c mice with intravenous injection of IO-PEG (2.5 mg Fe/kg) and Lp-IO (2.5 mg Fe/kg). Arrows (left to right) separately indicate water, kidney, liver and tumor. (*n* = 3.) (b) The normalized relative changes in T_2_-weighted MR signals in major tissues. (c) Schematic diagram of 4T1 tumor-bearing Balb/c mouse fabrication and treatments with Lp (20 mg/kg), IO-PEG (2.5 mg Fe/kg) and Lp-IO at different doses (L, 1 mg Fe/kg; H, 2.5 mg Fe/kg) (*n* = 5). Arrows indicate treatment. (d) Tumor volume variation in 14 days (*n* = 5). (e) T_2_-weighted MRI on the 15th day. Borders indicate tumor tissues. (f) Bodyweight variation of the mice (*n* = 5). (g) Perls blue, (h) H&E, Tunel and Ki67 (IHC) staining of the tumor tissues after treatment. Scale bars: 250 μm. *: compared with NC. *, *P* < 0.05; **, *P* < 0.01.

Considering the relatively high iron content in organisms, it is inappropriate to study the pharmacokinetics of Lp-IO based on the iron content. Although the accurate location of T_2_ contrast agents may be misidentified due to the blooming effect and the presence of other hypointense areas, non-intrusive and real-time MRI is still a better method to investigate the *in vivo* biodistribution and excretion of Lp-IO after intravenous injection. A T_2_-weighted MRI of tumor, liver and kidney tissues was enhanced by Lp-IO and IO-PEG (Fig. 3a). Pseudo-color T_2_-weighted MR images of tumors with high resolution are provided. The average T_2_-weighted MRI signal of each tissue was quantified via ImageJ software. The relative signal changes at time intervals were normalized to the maximum to reveal the accumulation of Lp-IO or IO-PEG in the tissues (Fig. 3b). The MRI signal decreased the most at 8th hour after the injection of Lp-IO, indicating that the accumulation of Lp-IO in the tumor, liver and kidney reached a maximum. Lp-IO was continuously cleared from the tumor and kidney after 12 h, and ∼25% and 50% of Lp-IO remained residual for >120 h, respectively. In contrast, IO-PEG mainly accumulated within 6 h and was rapidly cleared from the tumor and kidney within 8 h. Lp-IO had a longer residence time at the tumor site than IO-PEG and was slowly excreted by the liver, which is attributed to the enhanced permeability and retention (EPR) effect on Lp-IO with proper size distribution [35,36].

4T1 tumor-bearing Balb/c mice were used to evaluate the *in vivo* ferroptosis-associated antineoplastic effect of Lp-IO (Fig. 3c). The mice were intravenously injected with Lp (20 mg/kg), IO-PEG (2.5 mg Fe/kg) and Lp-IO at a low dose (L, 1 mg Fe/kg) and high dose (H, 2.5 mg Fe/kg) once a day for one week. According to the tumor volume variation, IO-PEG did not alleviate tumor growth compared with biocompatible Lp. At the same time, Lp-IO had inhibition rates of ∼23.4% and ∼66.2% at the low and high doses, respectively (Fig. 3d). In addition, T_2_-weighted MR images of the mice on the 14th day reveal that Lp-IO diminishes the tumor tissues with dose dependence (Fig. 3e). There was no significant weight difference between the groups (Fig. 3f), indicating the negligible side effects of Lp-IO at effective doses. As revealed by the Perls blue staining of Fe^3+^ ions, IONPs were accumulated in tumor tissues (Fig. 3g). Hematoxylin and eosin (H&E) and Tunel staining showed extensive tumor necrosis and apoptosis. The down-regulation of Ki67 expression, tested by immunohistochemistry (IHC), indicated tumor growth inhibition (Fig. 3h). Combining IONPs with the supply of endogenous and exogenous H_2_O_2_ or chemotherapeutic agents like cisplatin is an efficient strategy to enhance the antineoplastic effect of ferroptosis [37–39]. By comparing the effective cumulative iron dose, embedding IONPs in liposome bilayer got higher antineoplastic efficacy than the combination strategies. In addition, there was no visible damage to major organs, including the heart, liver, spleen, lung and kidney (Fig. S30). The raw materials of Lp-IO, including PEG, phospholipid, cholesterol and ultrasmall iron oxide, exhibit excellent biocompatibility and are slowly metabolizable [36]. Lp-IO has a unique antitumor ability via ferroptosis activation, and outstanding biosafety.

### pH/ROS-responsive release of drugs

**Figure 4.**
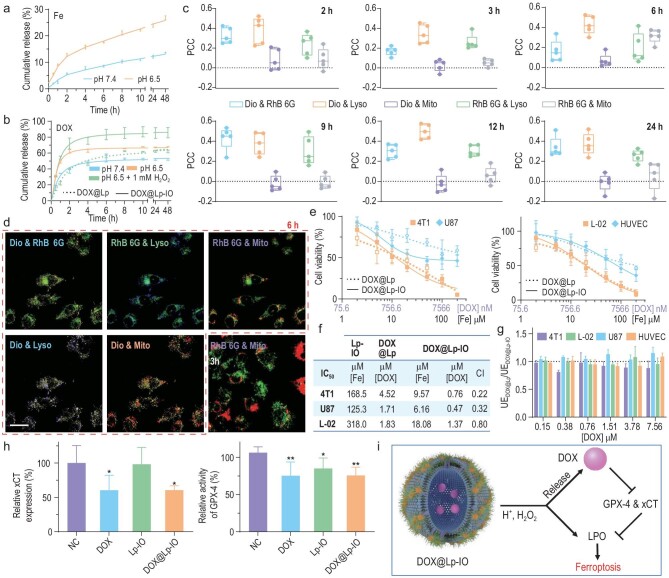
pH/ROS-responsive capability and synergistic antitumor effect with chemotherapy *in vitro*. (a) Cumulative release of iron ions from Lp-IO at pH 7.4 and 6.5 (*n* = 3). (b) Cumulative release of DOX from DOX@Lp-IO at pH 7.4 and pH 6.5 (± 1 mM H_2_O_2_) and from DOX@Lp at pH 6.5 (+ 1 mM H_2_O_2_) (*n* = 3). (c and d) 4T1 cells were treated with RhB6G@Lp-IO&Dio and stained with LysoTracker™ Blue and MitoTracker™ Deep Red (*n* = 5). (c) PCC analysis of the FL signals. (d) Merged FL images of 4T1 cells at 3 and 6 h; scale bar: 20 μm. (e) Viability of cancer cells (4T1 and U87) and normal cells (L-02 and HUVEC) treated with DOX@Lp and DOX@Lp-IO for 24 h. (f) IC_50_ and CI for Lp-IO, DOX@Lp and DOX@Lp-IO. Additive effect (CI = 1), synergism (CI < 1) and antagonism (CI > 1). (g) Relative cellular uptake of DOX@Lp and DOX@Lp-IO based on the intracellular DOX contents. (h) Relative xCT expression and GPX-4 activity in 4T1 cells treated with PBS (NC), DOX, Lp-IO or DOX@Lp-IO (*n* = 3). (i) Synergistic mechanism of Lp-IO and DOX released from DOX@Lp-IO. *: compared with NC. *, *P* < 0.05; **, *P* < 0.01.

Liposomes are FDA-approved nanosized carriers with an internal water phase for hydrophilic drugs and a phospholipid bilayer for hydrophobic drugs. Lp-IO can induce ferroptosis via IO-PEG dissociation and lipid bilayer peroxidation under acidic and oxidative conditions, probably making it pH/ROS-responsive and ferroptosis-activating cargo for drug delivery. To evaluate the on-pH/ROS release capability and synergistic effect of ferroptosis and chemotherapy, DOX was encapsulated into the internal phase of Lp-IO (DOX@Lp-IO) and Lp (DOX@Lp) for comparison. DOX@Lp-IO was prepared with a mass ratio of iron to DOX of 1 : 0.73. The cumulative release of Fe^3+^ ions from Lp-IO at pH 6.5 was almost twice that at pH 7.4 (Fig. 4a), indicating the on-acidity dissociation of IO-PEG NPs. DOX@Lp-IO had a higher DOX release rate at pH 6.5 than pH 7.4, and it was further improved in the presence of H_2_O_2_. Under pH 6.5 and the presence of H_2_O_2_, DOX@Lp had a DOX release rate comparable to that of DOX@Lp-IO at pH 7.4 (Fig. 4b). Most DOX was released in 6 h, and the cumulative release rates were 52.9%, 63.9% and 83.3% for DOX@Lp-IO at pH 7.4, pH 6.5 and pH 6.5 with the presence of H_2_O_2_, respectively. This value was 56.4% for DOX@Lp at pH 6.5 with the presence of H_2_O_2_. Compared with DOX@Lp, DOX@Lp-IO improves DOX release by 50% and enables a high release rate under acidic and oxidative conditions, indicating that Lp-IO is pH/ROS dual responsive for drug delivery.

In addition, to monitor the fate and drug delivery behaviors of Lp-IO in cancer cells, rhodamine 6G (RhB 6G) targeting mitochondria and 3,3′-dioctadecyloxacarbocyanine perchlorate (Dio) as a lipophilic tracer were separately loaded in the internal phase and the bilayer of Lp-IO to prepare fluorescent RhB6G@Lp-IO&Dio. Lysosomes and mitochondria were individually labeled with LysoTracker™ Blue and MitoTracker™ Deep Red. The four fluorescent probes were carefully selected according to their excitation and emission spectra (Figs S31 and S32) to avoid mutual interference of fluorescent signals in confocal laser microscopic imaging. 4T1 cells were treated with RhB6G@Lp-IO&Dio at different time intervals, and all FL images were captured and analyzed under the same conditions (Fig. S33). Pearson's colocalization coefficient (PCC) based on Costes’ automatic threshold, a unitless parameter for colocalization analysis of FL signals [40], was obtained in designated regions of interest (ROIs) with ImageJ software (Fig. 4c). The PCC of Dio and Lyso (LysoTracker™ Blue) was much higher than that of Dio and Mito (MitoTracker™ Deep Red), indicating the distribution of RhB6G@Lp-IO&Dio in lysosomes rather than mitochondria after cell uptake. The PCC values of RhB6G and Lyso were higher than those of RhB6G and Mito at different time intervals except for 6 h, also revealing the preferred location of RhB6G@Lp-IO&Dio in lysosomes. Notably, the PCC of RhB 6G and Mito increased suddenly at 6 h and returned to a low level at 9 h. Corresponding to the release behavior of DOX@Lp-IO, RhB 6G should be released from RhB6G@Lp-IO&Dio within 6 h and rapidly enriched in mitochondria via passive targeting. Subsequently, the RhB 6G efflux from mitochondria was continuous due to its reduced cytoplastic levels, attributed to excessive cellular excretion by P-glycoprotein [41]. The colocalization analysis was visually confirmed with corresponding merged FL images (Figs 4d and S33). The FL of RhB 6G and MitoTracker only overlapped at 6 h, while it was negligible at other times. Therefore, Lp-IO is enriched in acidic lysosomes after cell uptake, promoting on-pH cleavage for controlled drug release.

### Synergy of ferroptosis and chemotherapy by DOX@Lp-IO

Classic chemotherapeutic drugs (e.g. DOX, cisplatin and paclitaxel) have a synergistic effect with ferroptosis by two parallel systems: GSH/GPX-4 and ferroptosis suppressor protein 1 (FSP1)/ubiquinone (CoQ10)/NAD(P)H axes, resulting in the blockage of LPO reduction [4,42,43]. In addition to GSH/GPX-4 inactivation, DOX commonly activates P53 [44,45], which can suppress the SLC7A11 gene (xCT) to alleviate GSH synthesis. Chemotherapeutic drugs loaded with Lp-IO, such as DOX@Lp-IO, probably achieve synergy in cancer therapy. Compared with DOX@Lp, DOX@Lp-IO enhanced the inhibitory effect in cancer cells (Fig. 4e). The IC_50_ of DOX decreased from 4.52 μM to 0.76 μM in 4T1 cells and from 1.71 μM to 0.47 μM in U87 cells (Fig. 4f), indicating that Lp-IO delivery improved the sensitivity of cancer cells to DOX. In contrast, DOX@Lp-IO and DOX@Lp had comparable inhibitory efficacy in normal L-02 and HUVEC (human umbilical vein endothelial cell line) cells, and the IC_50_ of DOX was 1.37 and 1.83 μM in L-02 cells, respectively. Through the combination index (CI) theorem of Chou-Talalay [46], DOX@Lp-IO showed a higher synergism in 4T1 (0.22) and U87 (0.32) cells than in L-02 (0.80) cells, indicating selective synergy in tumor cells. In addition, the uptake efficiency (UE) of DOX@Lp and DOX@Lp-IO, as determined via quantification of cellular DOX, was almost the same in these cells (Fig. 4g), indicating that Lp-IO indeed enhanced chemosensitivity instead of improving cell uptake of DOX. When 4T1 cells were treated with DOX or DOX@Lp-IO, the intracellular xCT level decreased by 39.5% and 39.4%, and the GPX-4 activity dropped by 24.7% and 24.3% (Fig. 4h), respectively. Therefore, DOX@Lp-IO improves the cumulative release of DOX in cancer cells, and ‘free’ DOX enhances Lp-IO-induced ferroptosis via GPX-4 inactivation and xCT inhibition, resulting in the synergistic antitumor effect of ferroptosis and chemotherapy (Fig. 4i). Besides, DOX can activate nicotinamide adenine dinucleotide phosphate (NADPH) oxidase (NOX) to enhance the NOX/NADPH/O_2_/O_2_^−^/H_2_O_2_ system for ferroptosis [47].

**Figure 5.**
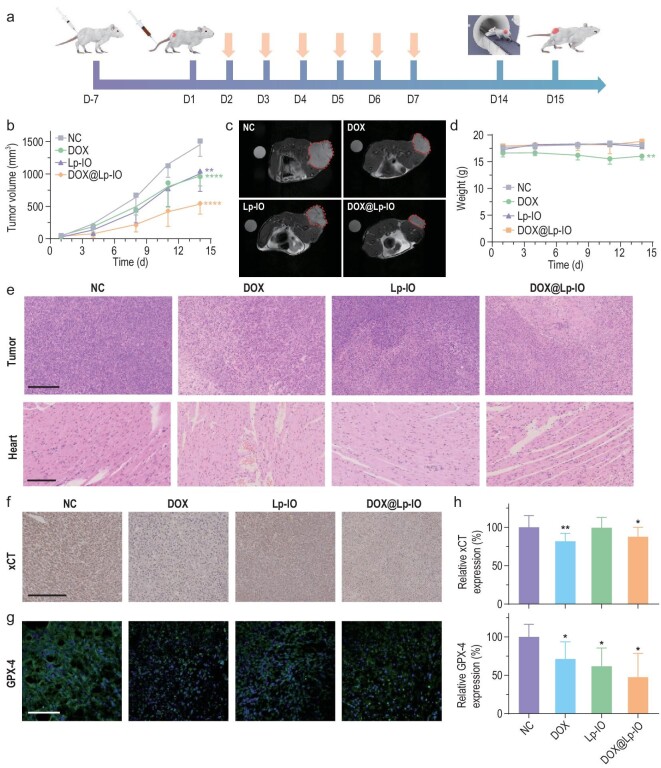
*In vivo* antineoplastic effect of DOX@Lp-IO. (a) Schematic diagram of 4T1 tumor model fabrication and treatments with saline (NC), DOX (2.5 mg/kg), Lp-IO (Fe, 1 mg/kg) and DOX@Lp-IO (DOX, 2.5 mg/kg; Fe, 1 mg/kg) (*n* = 5). The arrows indicate treatment. (b) Tumor volume variation (*n* = 5). (c) T_2_-weighted MR images of tumor-bearing mice on the 18th day. Tumor tissues are labeled with borders. (d) Bodyweight variation of the mice (*n* = 5). (e) H&E staining of tumor and heart after treatment (*n* = 5); scale bars: 250 μm. The IHC staining of (f) xCT and (g) GPX-4 in tumor tissues and (h) the corresponding quantification (*n* = 5); scale bars: 100 μm. *: compared with NC. *, *P* < 0.05; **, *P* < 0.01; ***, *P* < 0.001; ****, *P* < 0.0001.

The *in vivo* antineoplastic effect of DOX@Lp-IO was evaluated in 4T1 tumor-bearing mice via intravenous injection. DOX@Lp-IO was prepared with a mass ratio of iron to DOX of 1 : 2.5. Similar to Lp-IO, DOX@Lp-IO also accumulated in the liver, kidney and tumor within 8 h and was then slowly metabolized by the liver and kidney according to the MRI analysis (Fig. S34). The mice were treated with saline (NC), DOX (2.5 mg/kg), Lp-IO (Fe, 1 mg/kg) and DOX@Lp-IO (DOX, 2.5 mg/kg; Fe, 1 mg/kg) once a day for one week (Fig. 5a). The inhibition rates based on tumor volume variation were 36.3%, 30.8% and 63.9% for DOX, Lp-IO and DOX@Lp-IO, respectively (Fig. 5b). The synergistic antineoplastic effect of DOX and Lp-IO was significant, and the CI was ∼1.22. Tumor growth inhibition was visually confirmed with MRI (Fig. 5c) and photographs of the dissected tumor tissues (Fig. S35). Correspondingly, the inhibition rates evaluated by tumor weight changes (DOX, 34.5%; Lp-IO, 34.7%; DOX@Lp-IO, 68.3%) were almost identical to those calculated from tumor volume variations (Fig. S36). Notably, DOX treatment caused weight loss of more than 10% (Fig. 5d), and H&E-stained cardiomyocytes showed disordered arrangements and enlarged gaps (Fig. 5e), revealing the significant cardiotoxicity of DOX. H&E-stained tumor slices showed extensive necrosis after DOX@Lp-IO treatment. In contrast, negligible weight loss and invisible damage in pathological slices of major tissues, including the heart, were observed for DOX@Lp-IO at the same dose as the DOX group (Fig. S37), indicating that Lp-IO alleviated the toxicity of DOX and improved chemosensitivity. The expression of xCT and GPX4 in tumor tissues were evaluated by IHC and quantified for statistical analysis (Fig. 5f and g). The xCT expression was down-regulated by 18.2% and 12.3% by DOX and DOX@Lp-IO, respectively (Fig. 5h). The cellular GPX-4 levels dropped by 28.8%, 38.3% and 52.5% after treatments with DOX, Lp-IO and DOX@Lp-IO, respectively. Therefore, DOX@Lp-IO inhibits GPX-4 activity and xCT to improve ferroptosis sensitivity, further enhancing chemotherapy responses *in vivo*.

## CONCLUSION

Ferroptosis activation can abrogate resistance to chemotherapeutic and targeted agents. Increasing the intrabilayer ROS content for efficient initiation of lipid peroxidation is a promising alternative for triggering ferroptosis. Therefore, we designed Lp-IO by embedding PEG-PO-modified 3 nm γ-Fe_2_O_3_ NPs into the bilayer of liposomes containing ULs. In the membranes of Lp-IO, γ-Fe_2_O_3_ NPs converted diffusion-limited H_2_O_2_ to •OH/•OOH through the Fenton reaction, which rapidly reacted with the neighboring ULs to magnify LPO production and subsequently induced ferroptosis for cancer therapy *in vitro* and *in vivo*. MD simulations revealed that the amphiphilic PEG moieties on γ-Fe_2_O_3_ NPs integrated into the liposomal bilayer to improve the membrane permeability to H_2_O_2_ and •OH, for sufficient intrabilayer supply. In addition, Lp-IO integrates the capabilities of MRI tracing and pH/ROS dual-responsive drug delivery. DOX, which inhibits xCT and GPX-4, was delivered by Lp-IO, resulting in the synergistic antineoplastic effect of ferroptosis and chemotherapy, and significantly reduced toxicity. This work provides an efficient strategy to initiate lipid peroxidation for ferroptosis and a novel drug delivery vehicle for combination therapies in cancer.

## Supplementary Material

nwac167_Supplemental_Files
