# Supplementary material for: Liposomes embedded with PEGylated iron oxide nanoparticles enable ferroptosis and combination therapy in cancer
Source: Natl Sci Rev. 2022 Aug 18;10(1):nwac167. doi: 10.1093/nsr/nwac167 (PMC9843134; doi:10.1093/nsr/nwac167)
Supplement: nwac167_Supplemental_Files [file nwac167_supplemental_files.zip › Supplementary_data.pdf]

Liposomes Embedded with PEGylated iron oxide nanoparticles enabling ferroptosis and combination therapy in cancer

Yang Liu[a,b,#], Xuebo Quan[d,#], Jie Li[a,*], Jiawei Huo[a,b], Xing Li[b,c], Zhongpu Zhao[a,b], Shumu Li[a], Jing Wan[a,b], Jiao Li[a], Shuai Liu[a], Tao Wang[a], Xing Zhang[a], Bo Guan[a], Rui Wen[a], Zhenwen Zhao[b,c], Chunru Wang[a,b,*] and Chunli Bai[a,b,*]

[a]Beijing National Research Center for Molecular Sciences, Key Laboratory of Molecular Nanostructure and Nanotechnology, Institute of Chemistry, Chinese Academy of Sciences, Beijing 100190, China;

[b]University of Chinese Academy of Sciences, Beijing 100049, China;

[c]Key Laboratory of Analytical Chemistry for Living Biosystems, Institute of Chemistry Chinese Academy of Sciences, Beijing Mass Spectrum Center, Beijing 100190, China;

[d]Institute of Systems and Physical Biology, Shenzhen Bay Laboratory, Shenzhen 518107, China

*__Corresponding authors__. E-mails: clbai@cas.cn; crwang@iccas.ac.cn; lijie24@iccas.ac.cn

[#]Equally contributed to this work.

This PDF file contains:

Methods, Figures S1-S38, and Table S1

METHODS

Materials and Reagents. Methoxypolyethylene glycol (M_w 1900) and phosphorus oxychloride were purchased from Alfa Aesar. Lecithin from Soybean and cholesterol were purchased from Sinopharm Chemical Reagent Beijing Co., Ltd. 1,2-distearoyl-sn-glycero-3-phosphoethanolamine-N-[amino(polyethylene glycol)-2000] (DSPE-PEG), sodium oleate, iron(III) chloride hexahydrate, oleic acid, oleyl alcohol, diphenyl ether, 5, 5-Dimethyl-1-pyrroline N-oxide, amiloride hydrochloride, chlorpromazine hydrochloride, and cytisine were purchased from Sigma-Aldrich. Doxorubicin hydrochloride was purchased from Beijing Innochem Science & Technology CO., Ltd. 1-lauroyl-2-hydroxy-sn-glycero-3-phosphocholine (12:0 LPC) and DPPC (16:0 PC) were purchased from Avanti Polar Lipids. Antibodies for western blot and immunohistochemistry assay were purchased from Abcam. All reagents and solvents were used directly without further purification.

Preparation. PO-PEG, IONPs of 2 nm and 3 nm, and IO-PEG were synthesized according to the reported works [1]. For the Lp-IO preparation, 40 mg lecithin, 10 mg cholesterol, 5 mg DSPE-PEG, and 8.5 mg IO-PEG containing 3 mg Fe were dissolved in the mixed solvent of 10 mL dichloromethane and 10 mL methanol. The solvent was removed with a rotary evaporator at 42 ℃ to obtain a brown translucent film. The film was stripped with 8 mL water and broken at 700 W for 10 min in an ice bath. Iron residues from the ultrasound probe were precipitated by centrifugation at 5000 rpm for 10 min. The unloaded IO-PEG were removed with C18 spherical silica gel chromatographic packing (SP-300-5-ODS-B10, DAISO). The purified Lp-IO was filtered by 0.22 μm film, freeze-dried, and stored at 4 ℃. Following the preparation procedure of Lp-IO, Lp was prepared without addition of IO-PEG, and UL-free Lp-IO was obtained by replacing lecithin with 40 mg DPPC (16:0 PC). For Lp-IO&Dio, DOX@Lp-IO, and RhB 6G@Lp-IO&Dio, 1 mg Dio, 5 mg DOX, and 1mg Dio and 1 mg RhB 6G were added in the preparation, respectively.

Characterization. Morphology images of IONPs and IO-PEG were captured by a TEM (JOEL JEM-2100) at 100 kV. ^{1}H, ^{13}C, and ^{31}P NMR spectra of PEG and

PEG-PO were obtained by nuclear magnetic resonance spectroscopy (AVANCE 400 and AVANCE III 500WB, Bruker). Morphology images of Lp, Lp-IO, and UL-free Lp-IO were captured by cryogenic electron microscopy (Themis 300, FEI) at 200 kV. The magnetic force microscopy (MFM) measurements were performed using an atomic force microscope (Bruker Corp., Dimension Icon) in an Ar-filled glovebox. MFM images were achieved using a magnetic HA_FM/CoFe tip (77 kHz resonance frequency, radius < 35 nm, k=3.5 N/m, CoFe coating, NT-MDT & TipsNano Corp.) to scan the surface of Lp, Lp-IO, and Lp mixed with IO-PEG in MFM mode. The hydrodynamic size and zeta potential were measured by a dynamic light scattering (ZEN 3600 Zeta sizer Nano ZS, Malvern). The iron concentrations were measured with an ICP-MS (NexION 300X, PerkinElmer). XPS was obtained by a multifunctional photoelectron spectrometer (ESCALAB250XI, Thermo Fisher Scientific). XRD was performed with an X-ray diffractometer (Panaco Empyrean). Magnetic hysteresis loops were obtained by a vibrating sample magnetometer (VSM, 7404/21700GS, Lake Shore).

Hydroxyl radical assay. The hydroxyl radicals were captured by DMPO and detected with an electron spin resonance spectrometer (ESR, JEOL FA-200, JEOL, Japan). 100 μL DMPO (100 mM) and 50 μL H_2O_2 solution (5 mM) were mixed with 50 μL of ultrapure water, Lp (50 μg/mL), IO-PEG (Fe 20 μg/mL), and Lp-IO (Fe 20 μg/mL), respectively. The X-band EPR spectra of DMPO-OH were recorded in the dark with parameters: microwave frequency = 9.054 GHz, microwave power = 0.998 mW, modulation frequency = 100.00 kHz, and modulation amplitude = 2.00 G.

LPO assay. LPO was stained by C11-BODIPY (Thermo Fisher) and measured by an iMark microplate reader (Bio-RAD, USA). C11-BODIPY (1 μM) in black 96-well microplate was mixed with the following: Lp (480 μg/mL), Lp (480 μg/mL) with 1 mM H_2O_2, Lp-IO (500 μM Fe), Lp-IO (500 μM Fe) with 1 mM H_2O_2, Lp (480 μg/mL) with IO-PEG (500 μM Fe), and Lp (480 μg/mL) with IO-PEG (500 μM Fe) and 1 mM H_2O_2. And the LPO production was evaluated by the ratio of fluorescence intensities in FL1 (λ_{ex} = 488 nm, λ_{em}= 510 nm) and FL2 (λ_{ex} = 581 nm, λ_{em}= 610 nm) channels.

Identification and quantification of lipid and LPO. The samples were separately

incubated for 6 h at 37 ℃ as follows: Lp (480 μg/mL), Lp (480 μg/mL) with 1 mM H_2O_2, Lp-IO (500 μM Fe), Lp-IO (500 μM Fe) with 1 mM H_2O_2, Lp (480 μg/mL) with IO-PEG (500 μM Fe), and Lp (480 μg/mL) with IO-PEG (500 μM Fe) and 1 mM H_2O_2. Lipid and LPO molecules were detected by the previous method [2]. In short, 100 μL of samples were extracted with 900 μL methanol (1.11 μM 12:0 LPC as an internal standard), vortexed for 5 min, and centrifuged at 10000 rpm. 10 μL supernatant was separated by a ultra-performance liquid chromatography (I-class Acquity, Waters) with parameters as follows: column (ACQUITY UPLC@BEH C18), mobile phase A (isopropanol/acetonitrile/formic acid = 90:10:0.1 with 10 mM ammonium formate), mobile phase B (acetonitrile/water/formic acid = 70:30:0.1 with 10 mM ammonium formate), column temperature (55℃), flow rate (0.3 mL/min), total time (20 min), elution gradient (0 min 70% B, 2 min 57% B, 2.1 min 50% B, 12 min 46% B, 12.1 min 30% B, 18 min 1% B, 18.1 min 70% B, 20 min 70% B). Mass spectrometry was further analyzed using a mass spectrometer (API 4500 QTRAP, Applied Biosystems/MDS SCIEX) with parameters as follows: positive ion MRM mode, curtain gas (20), collision gas medium, ion source gas 1 (40), ion source gas 2 (40), electrospray voltage (5000), temperature (550 ℃), declustering potential (130), entrance potential (10), collision energy (35), and collision cell exit potential (13). The peak areas of lipid and LPO molecules were analyzed by Analyst 1.6 software. The ULs undergoing significant peroxidation were quantified and screened with several inclusion criteria: ⩾10-fold signal-to-noise ratio; ⩾2.5-fold increase in the relative peak area ratio of doubly oxygenated lipid (LOOH) to 12:0 LPC (internal standard) in the presence versus absence of H_2O_2 (P-value < 0.05); ⩾0.5% contribution to the LC-MS peak areas of liposomal phospholipids.

Molecular dynamics simulations. MD simulations were conducted to investigate the interactions between H_2O_2/•OH and lipid bilayers without or with embedded IO-PEG. In this work, DPPC, as the major lipid component in cell membranes, was chosen to construct a simplified lipid bilayer with dimensions of 8.7×8.7 nm (with 318 DPPC lipids). The diameter of IO particle is about 2 nm and decorated with 30 PEG chains (each chain consists of 10 monomers). The constructed IO-PEG was then

inserted into the pure lipid bilayer. The force field parameter for IO particle was adopted from Wand's work [3], and other potential parameters were taken from the widely used CHARMM force field [4]. Water molecules were represented by the SPC/E model. For each lipid bilayer system, a 100 ns MD simulation was performed to make them reach equilibration state in solution. After that, several H_2O_2/•OHs were placed above the equilibrated lipid bilayer with a distance of 2 nm, as shown in Figs S14 and S16. First, the simulation system was minimized by the steepest-descent method to eliminate the steric overlap or inappropriate geometry. Then, the lipid bilayer and H_2O_2/•OHs were constrained to equilibrate water for 10 ns. Finally, the constraints were removed, and a 100 ns MD production simulation was performed to achieve equilibration. The simulation trajectory was saved every 10 ps with a time step of 2.0 fs. All simulations were carried out under the semi-isotropic NPT ensemble with the GROMACS package (version 2019.3) [5]. The temperature was controlled at 310 K via the Berendsen thermostat with a relaxation time of 1 ps. A semi-isotropic Parrinello-Rahman barostat was adopted to couple the lateral and perpendicular pressures at 1 bar with a time constant of 5 ps. The non-bonded interactions were calculated at a cutoff distance of 1.2 nm. The particle mesh Ewald (PME) method with a cutoff radius of 1.2 nm was adopted to deal with electrostatic interactions. The Visual Molecular Dynamics (VMD) program was used for structure visualization [6]. By combining the steered MD (SMD) simulations and umbrella sampling method, the potential of mean force (PMF) profiles for the membrane permeation of a single H_2O_2/•OH were computed. First, the H_2O_2/•OH was pulled along the z-axis (perpendicular to the lipid bilayer) from the outside bulk solution to the membrane inside with a pull rate of 0.01 nm ps^{-1}. From the SMD simulation, a series of initial configurations with a 0.1 nm step size were generated for the following umbrella sampling simulations. A biased harmonic potential with force constant of 1000 kJ mol^{-1} nm^{-2} was used to confine the H_2O_2/•OH within the sampling window. Each window was simulated up to 10 ns under the NPT ensemble. The former 5 ns of each run were discarded, and the latter 5 ns were chosen for data analysis. In this way, the unbiased probability distribution functions can be obtained

using the weighted histogram analysis method (WHAM) to construct the PMF profile. The permeability coefficients for H_2O_2 and •OH were then calculated following the method reported before [7].

ROS and LPO assays in vitro. Intracellular ROS and LPOs were separately labeled with DCFH-DA and C11-BODIPY and detected by a flow cytometer (Olympus FV 1000-IX81, Japan). A549 and 4T1 cells were seeded into 12-well plates (1×10^5 cells per well), incubated for 12 h, and then treated as follows: with Lp (300 μg/mL), IO-PEG (300 μM Fe), Lp-IO (300 μM Fe), and RSL3 (5 μM, Ark) for 2, 4, and 6 h; with Lp (500 μg/mL), Lp-IO (100, 300, and 500 μM Fe), and UL-free Lp-IO (100, 300, and 500 μM Fe) for 6 h. Subsequently, the cells were separately incubated with 10 μM DCFH-DA (Sigma-Aldrich) and 5 μM C11-BODIPY (Thermo Fisher) for 30 min, and the flow cytometer counted the cellular FL (λ_{ex} = 488 nm, λ_{em} = 510 nm).

Cellular uptake mechanism of Lp-IO. 4T1 cells seeded in 96-well plates (5×10^3 cells per well) were separately pretreated with amiloride hydrochloride (0.4 μg/mL), chlorpromazine hydrochloride (0.4 μg/mL), and cytisine (40 μg/mL), and incubated with Lp-IO&Dio (100 μM Fe) for 6 h. The cells were lysed with RIPA Lysis Buffer (Beyotime), and the cellular Dio FL was measured (λ_{ex} = 488 nm, λ_{em} = 505 nm).

Cytotoxicity study of Lp-IO. 4T1, U87MG, and L-02 cells were seeded into 96-well microplates (5×10^3 cells per well) and incubated for 12 h. The cells were separately treated with the Lp, IO-PEG, Lp-IO, UL-free Lp-IO, and Lp+IO at gradient concentrations for 24 h and then cell counting kit-8 (CCK-8, Beijing Solarbio Science & Technology Co. Ltd) for 2 h. The cell viabilities were determined at 450 nm with a microplate reader.

Inhibition of ferroptosis. 4T1 cells seeded in 96-well plates (5×10^3 cells per well) were separately pretreated with Lip-1 (1 μM, Macklin) and Fer-1 (1 μM, Aladdin) and incubated with Lp-IO (150 μM Fe). The cell viabilities were measured with the CCK-8 after 24 h.

GPX-4 assay in vitro. 4T1 cells seeded in 12-well plates (1×10^6 cells per well) were treated with Lp (300 μg/mL), IO-PEG (300 μM Fe), and Lp-IO (300 μM Fe) for 9 h, respectively. The cells were lysed with RIPA Lysis Buffer (Beyotime) and then

centrifuged to collect the supernatant. The activity and expression of GPX-4 were evaluated with a glutathione peroxidase assay kit (Beyotime) and western blot (anti-GPX-4 antibody, Abcam).

TEM imaging of ferroptosis cells. 4T1 cells seeded in 100 mm culture dishes (5×10^6 cells per well) were treated with saline (NC), Lp (300 μg/mL), IO-PEG (300 μM Fe), and Lp-IO (300 μM Fe) for 24 h, respectively. The cells were digested, centrifuged, and fixed with glutaraldehyde (2.5%, EM Grade, Solarbio) for 4 h. Slices of the cells were prepared and observed with a transmission electron microscope (Tecnai Spirit, 120 kV).

MRI in vitro and in vivo. T_1-weighted and T_2-weighted relaxivities were measured with a 0.5 T MR scanner (NMI20-Analyst) and a 7.0 T MR scanner (Bruker Para Vision 6.0.1). The MRI was performed on the 7.0 T MR scanner. The IO-PEG and Lp-IO at Fe concentrations of 0, 0.2, 0.4, 0.6, 0.8, and 1 mM were placed in centrifuge tubes (0.5 mL) for MRI scanning, and the pseudo-color images were obtained with Image J software. Female BALB/c mice aged five weeks were ordered from Beijing HFK Bioscience CO., LTD, and subcutaneously inoculated with 2×10^6 4T1 cells. After 10 days, the tumor-bearing mice were randomly divided into two groups, separately injected (i.v.) with the IO-PEG (2.5 mg Fe/kg) and Lp-IO (2.5 mg Fe/kg), and then scanned by the 7 T clinical MRI scanner after 0, 0.5, 1, 2, 4, 6, 8, 12, 24, 48, 72, 96, and 120 h. The measurement parameters of the T_2-weighted FR-FSE sequence were as follows: TR = 791.59 ms, TE = 24 ms, and Slice thickness = 0.5 mm. The signal intensity of the NPs in the tumor, liver, and kidney was calculated by Image J software.

Antitumor effect of Lp-IO in vivo. 4T1 tumor-bearing female BALB/c mice were randomly grouped and treated with Lp (2.5 mg/kg), IO-PEG (2.5 mg Fe/kg), Lp-IO (low dose, 1 mg Fe/kg), and Lp-IO (high dose, 2.5 mg Fe/kg) via intravenous tail injection once a day for a week. The length and width of tumor tissues and the body weights were measured every day for 2 weeks. The tumor volume was calculated as the formula:

$$V=L\times W^2$$

Where V, L, and W represent the volume, length, and width of tumor tissues, respectively. T2-weighted MRI of the mice was performed on the 14th day. Finally, the mice were sacrificed by cervical dislocation to collect tumors and major organs for the following staining.

On-pH release of Fe^{3+} ions from Lp-IO. Lp-IO (45 μg Fe/mL, 1 mL) was packaged into dialysis bags (cutoff molecular weight of 3500 Da) and immersed into the PBS buffers (20 mL) of pH 7.4 and pH 6.5. The dialysate of 5 mL was collected at 0, 0.5, 1, 2, 4, 6, 8, 10, 24, and 48 h, and 5 mL of PBS was replenished. The dialysate was treated with 5% nitric acid and analyzed with the ICP-MS to measure the Fe^{3+} concentration. The cumulative release (CR_n) ratio was calculated according to the following formula:

$$CR_n(\%)=\frac{21c_n+5\sum_{i=1}^{n-1}c_i}{m_{Fe}}\times 100\%$$

Where c_n and m_{Fe} represent the Fe^{3+} concentration in the dialysate and the initial iron mass of Lp-IO (45 μg).

On-pH/ROS release of DOX from DOX@Lp-IO. The release of DOX was monitored by the same method. DOX@Lp and DOX@Lp-IO were packaged in dialysis bags and immersed in pH 7.4 and pH 6.5 (± 1 mM H_2O_2). The dialysate was collected at 0, 0.5, 1, 2, 4, 6, 8, 10, 24, and 48 h, respectively. The DOX concentration in the dialysate was quantified by a high-performance liquid chromatography (LC2030C 3D, Shimadzu) with parameters as follows: Agilent Eclipse XDB-C18 (column), acetonitrile: 1% phosphoric acid = 3:7 (mobile phase), 310 nm (detection wavelength), 35℃ (column temperature), 1 mL/min (flow rate), 20 min (total time), and 3.89 min (peak time). The cumulative release (CR_n) rate of DOX was calculated according to the following formula:

$$CR_n(\%)=\frac{21c_n+\sum_{i=1}^{n-1}c_i}{m_{DOX}}\times 100\%$$

Where c_n and m_{DOX} represent the DOX concentration in the dialysate and the initial DOX mass in DOX@Lp and DOX@Lp-IO (35 μg).

Drug delivery behaviors of Lp-IO inside cells. 4T1 cells seeded in confocal glass dishes (5×10^3 cells per well) were incubated with RhB 6G@Lp-IO&DiO for 2, 3, 6, 9, 12, and 24 h, respectively. The cells were washed 3 times with PBS and separately treated with 1 μM of LysoTracker™ Blue DND-22 (Invitrogen™) and MitoTracker™ Deep Red FM (Invitrogen™) for 0.5 h. The cells washed 3 times with PBS and incubated with 1mL of fix solution (Boyetime) for 10 min were imaged by a confocal microscope (Olympus) with the same parameters as follows: Lyso Blue (λ_{ex} = 405 nm, λ_{em}= 410-450 nm), DiO (λ_{ex} = 488 nm, λ_{em}= 495-505 nm), RhB 6G (λ_{ex} = 559 nm, λ_{em}= 565-595 nm), and Mito Deep Red (λ_{ex} = 635 nm, λ_{em}=650-700 nm). The PCC values of two FL probes were calculated in designated regions of interest (ROIs) that limit analysis on individual cells with ImageJ.

Cytotoxicity and uptake of DOX@Lp-IO. 4T1, U87, L-02, and HUVEC cells seeded in 96-well microplates (5×10^3 cells per well) were treated with DOX@Lp-IO and DOX@Lp at gradient concentrations for 24 h, and the cell viabilities were quantified with CCK-8. The combination index (CI) was calculated according to the following formula:

$$CI=\frac{c_{c,\,a}}{c_a}+\frac{c_{c,b}}{c_b}$$

Where c_a and c_b represent the DOX and Fe concentration of DOX@Lp and Lp-IO at IC_{50} and $c_{c,\,a}$ and $c_{c,\,b}$ represent the DOX and Fe concentration of DOX@Lp-IO at IC_{50}. For the uptake assay, 4T1, U87, L-02, and HUVEC cells were incubated with DOX@Lp-IO and DOX@Lp for 9 h. The cell lysates were obtained to determine the FL intensity of DOX at an excitation wavelength of 480 nm and an emission wavelength of 580 nm.

Intracellular xCT assay. 4T1 cells seeded in 12-well plates (1×10^6 cells per well) were treated with NC, DOX (22.7 μM), Lp-IO (Fe, 300 μM), and DOX@Lp-IO (DOX, 22.7 μM; Fe, 300 μM) for 9 h, respectively. The cellular xCT expressions were tested with an ELISA kit for SLC7A11 (Wuhan Cloud-Clone Corp.).

Antitumor effect of DOX@Lp-IO in vivo. 4T1 tumor-bearing female BALB/c mice were randomly grouped and treated with saline (NC), DOX (2.5 mg/kg), Lp-IO (1 mg

Fe/kg), and DOX@Lp-IO (2.5 mg DOX/kg, 1 mg Fe/kg) via intravenous injection once a day for a week. The tumor volume and the bodyweight were monitored daily, and MRI was performed on the 14th day. The combination index was calculated according to the following formula:

$$\text{combination index}=\frac{\text{inh}_{\text{DOX}}\times\text{inh}_{\text{Lp-IO}}}{\text{inh}_{\text{DOX@Lp-IO}}}$$

where $\text{inh}_{\text{DOX@Lp-IO}}$, $\text{inh}_{\text{DOX@Lp-IO}}$, and $\text{inh}_{\text{DOX@Lp-IO}}$ represent the relative tumor volume rates of the DOX, Lp-IO, and DOX@Lp-IO groups to the NC group. And when combination index>1, it is synergism. Tumor tissues and major organs were collected for the following stainings.

Histological preparation, immunohistochemical, H&E, Perls blue, and Tunel staining analysis. The tissues fixed in 4% paraformaldehyde were embedded in paraffin and subsequently sectioned to 5 μm. In immunohistochemical analysis, the sections were mounted on glass slides and heated for 30 min at 65 ℃, and the slides were deparaffined and hydrated (xylene, 10 min, twice; 100% alcohol, 5 min, twice; 95% alcohol, 2 min; 80% alcohol, 2 min; 70% alcohol, 2 min; water, 5 min; PBS, 3 min, thrice). Then, the slides were blocked for 30 min, incubated with 0.01 M sodium citrate buffer in a microwave oven 5 times, washed with PBS 3 times, and incubated with 5% serum for 30 min. For the xCT and ki67 expressions, the slides after removing the serum were incubated with primary anti-GPX-4/Ki67 antibody (Abcam) at room temperature for 1 h and subsequently at 4 ℃ for 12 h. After removing the antibodies, the slides were incubated with anti-mouse IgG (Abcam) at 37 ℃ for 30 min and washed with PBS 5 times. The slides were stained with diaminobenzidine (Solarbio) for 5 min and hematoxylin (Solarbio) for 20 s, washed by PBS, and blocked with neutral balsam (Solarbio). For the xCT expressions, the slides were incubated with primary anti-xCT antibody (Abcam) at room temperature for 1 h and subsequently at 4 ℃ for 12 h, incubated with anti-mouse IgG H&L (FITC, Abcam) and DAPI (Solarbio) at 37 ℃ for 30 min, and blocked with neutral balsam. For H&E, Perls blue, and Tunel staining analysis, the sections were deparaffined and hydrated by the same procedures and stained according to the product manual of the kits

(Solarbio). The H&E, Perls blue, Tunel, GPX-4, and Ki67 stained slides were scanned by a scanning microtome (KF-PRO-005, KFBIO), and xCT stained slides were observed by a confocal laser scanning microscope (OLYMPUS FV1000-IX81, Olympus). The GPX-4 and xCT expressions were calculated according to the average signals via ImageJ software.

Statistical analysis. All the data are presented as mean ± standard deviations (SD). One-way analysis of variance (ANOVA) was used to analyze the difference between multiple groups by the software GraphPad Prism 8.2.1.

Ethical Statement. All animal experiments were conducted according to protocols approved by the Institutional Animal Care and Use Committee in Institute of Chemistry, Chinese Academy of Sciences (approval number is SYXK (Jing) 2018-0033).

Supplementary Figures

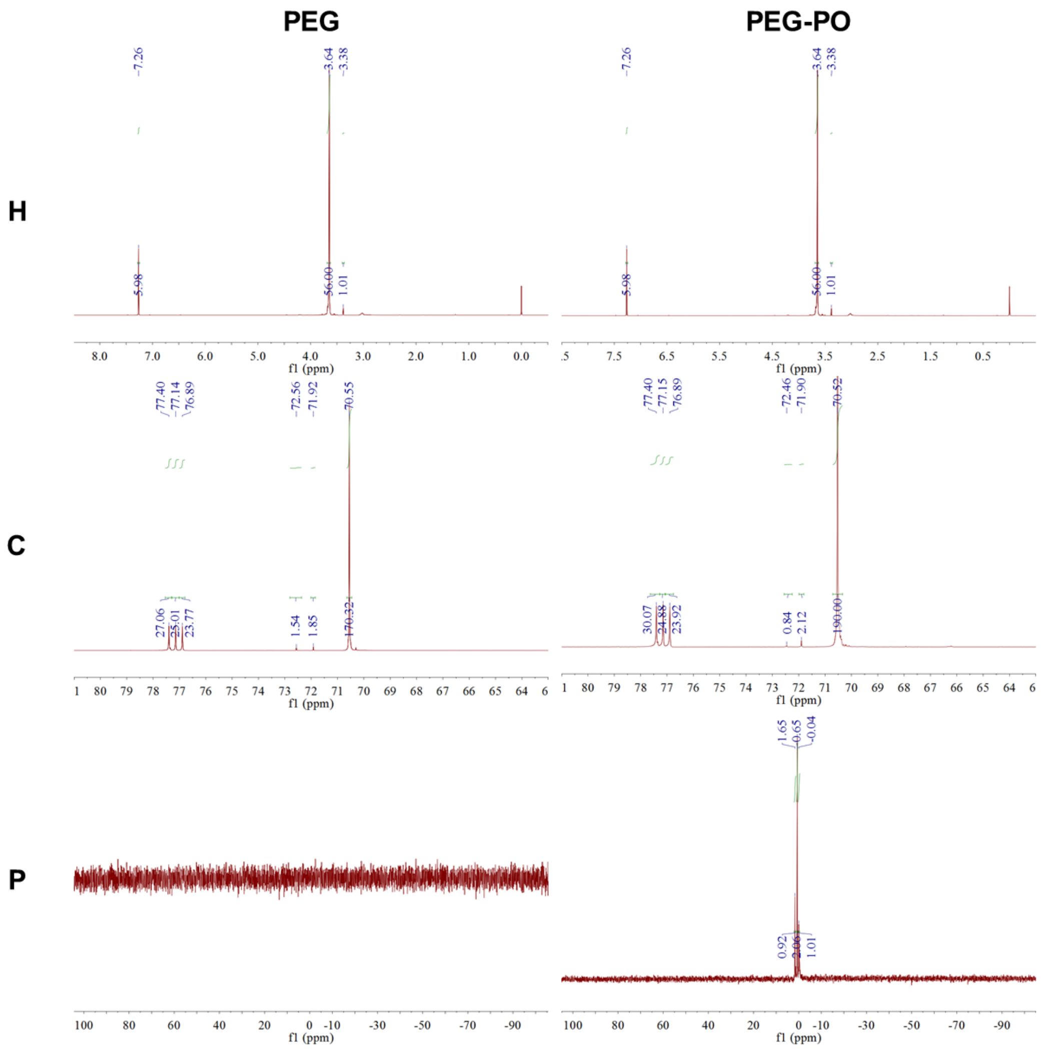

Figure S1. ^{1}H, ^{13}C, and ^{31}P NMR spectrum of PEG (M_w 1900) and PEG-PO.

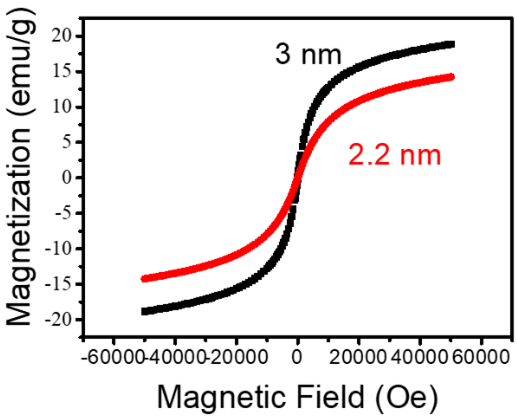

Figure S2. Magnetic hysteresis loops of 3 nm and 2.2 nm IONPs.

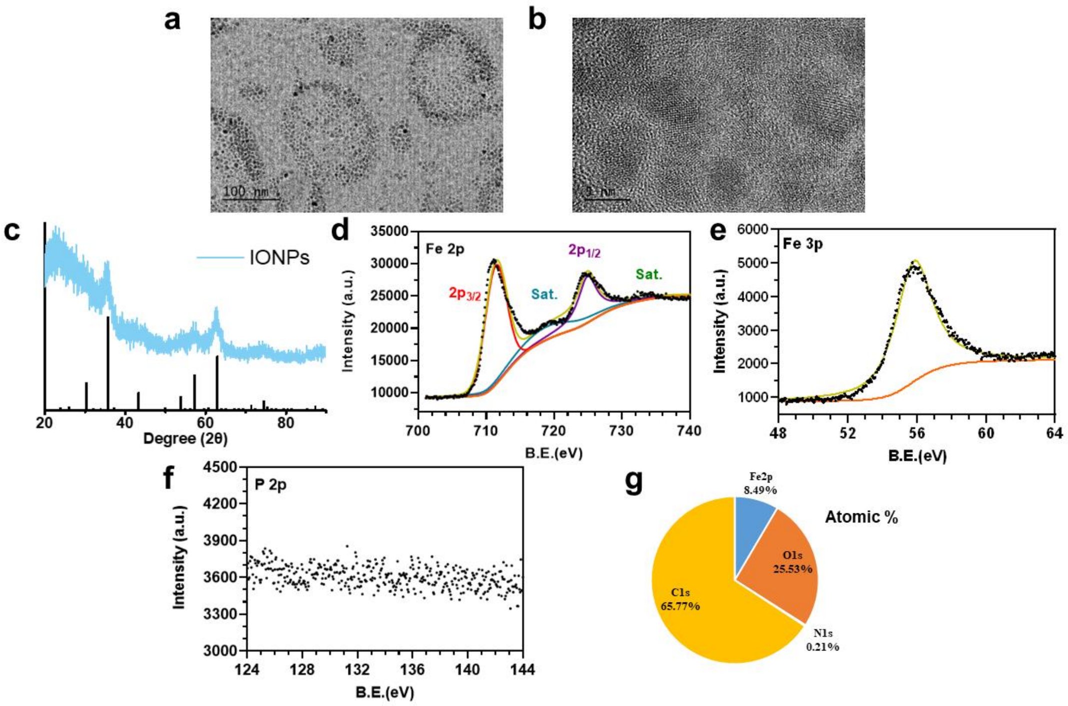

Figure S3. (a) TEM image of 3 nm IONPs. (b) High-resolution TEM images of 3 nm IONPs. (c) XRD patterns of IONPs and standard γ-Fe_2O_3. (d) Fe 2p, (e) Fe 3p and (f) P 2p XPS spectra of IONPs. (g) C, N, O, P, and Fe atomic ratio of IONPs.

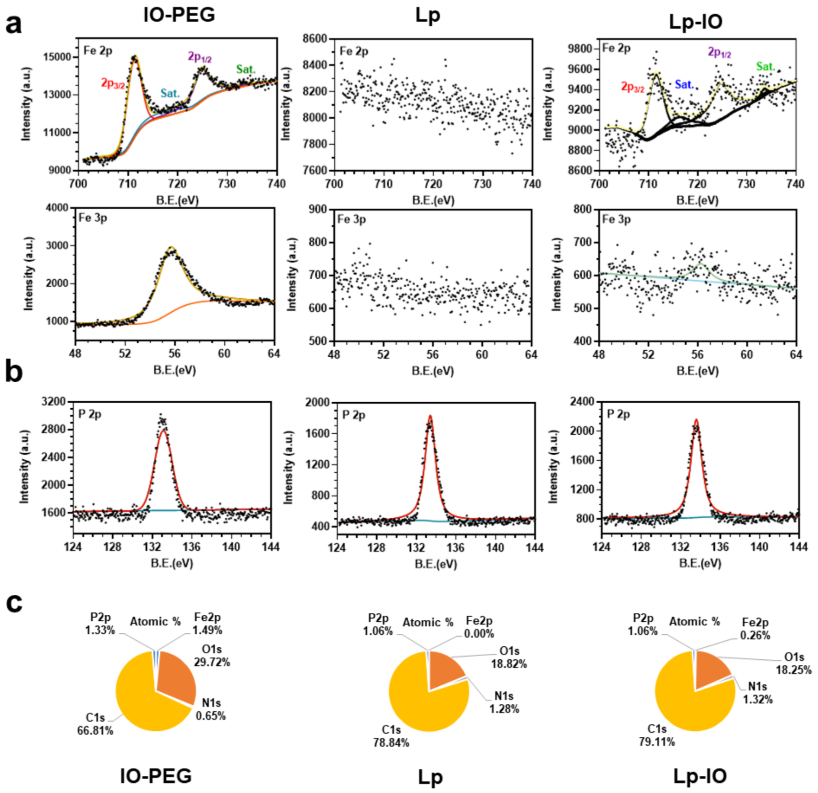

Figure S4. (a) Fe 2p and Fe 3p XPS spectra of IO-PEG, Lp, and Lp-IO. (b) P 2p XPS spectra of IO-PEG and Lp. (c) C, N, O, P, and Fe atomic ratio of IO-PEG, Lp, and Lp-IO.

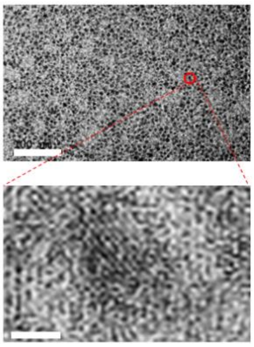

Figure S5. Top: TEM image of 3 nm IO-PEG; scale bar is 100 nm. Bottom: High-resolution TEM image of IO-PEG; scale bar is 2 nm.

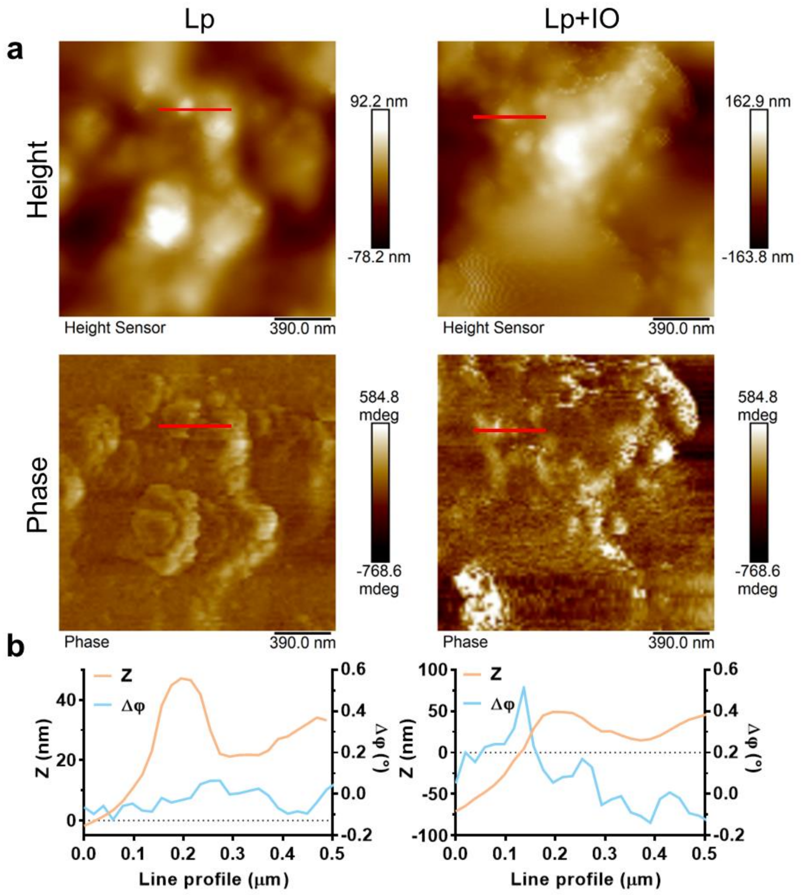

Figure S6. (a) AFM (top) and MFM (bottom) image of Lp and Lp mixed with IO-PEG. (b) Profile analysis of red lines.

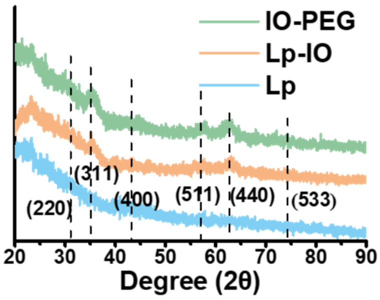

Figure S7. XRD patterns of IO-PEG, Lp, and Lp-IO.

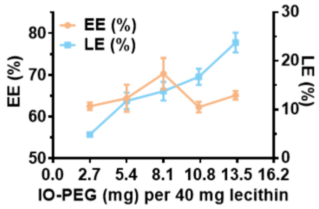

Figure S8. Encapsulation efficiency and loading efficiency of Lp for IO-PEG at different ratios of IO-PEG to Lp (consisting of 40 mg lecithin).

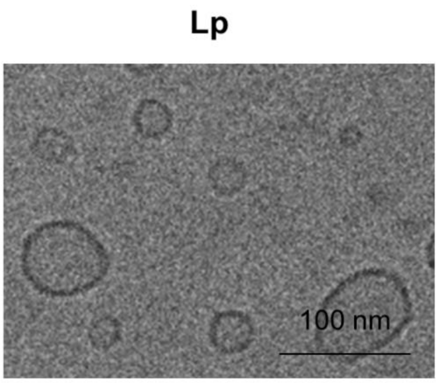

Figure S9. Cyro-TEM image of Lp.

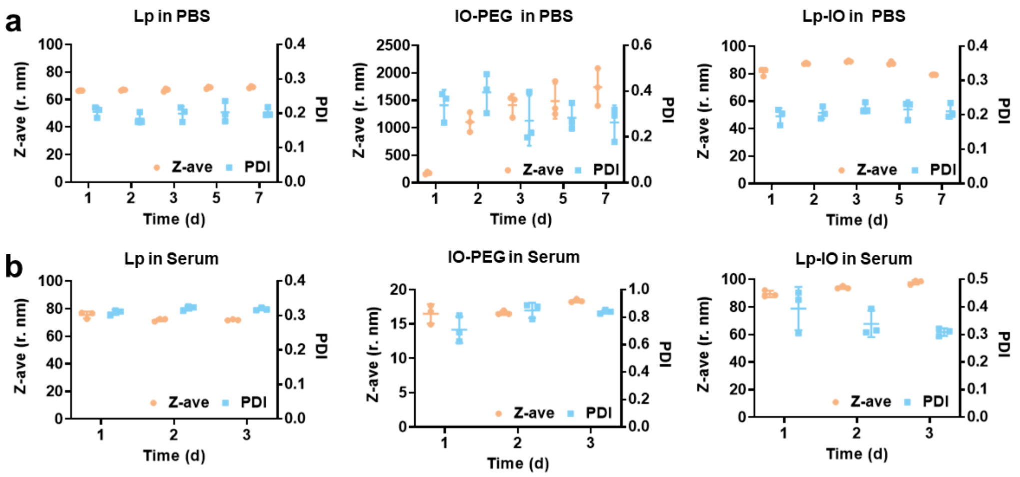

Figure S10. The stability of Lp, IO-PEG, and Lp-IO in PBS (a) and serum (b) at 37 ℃.

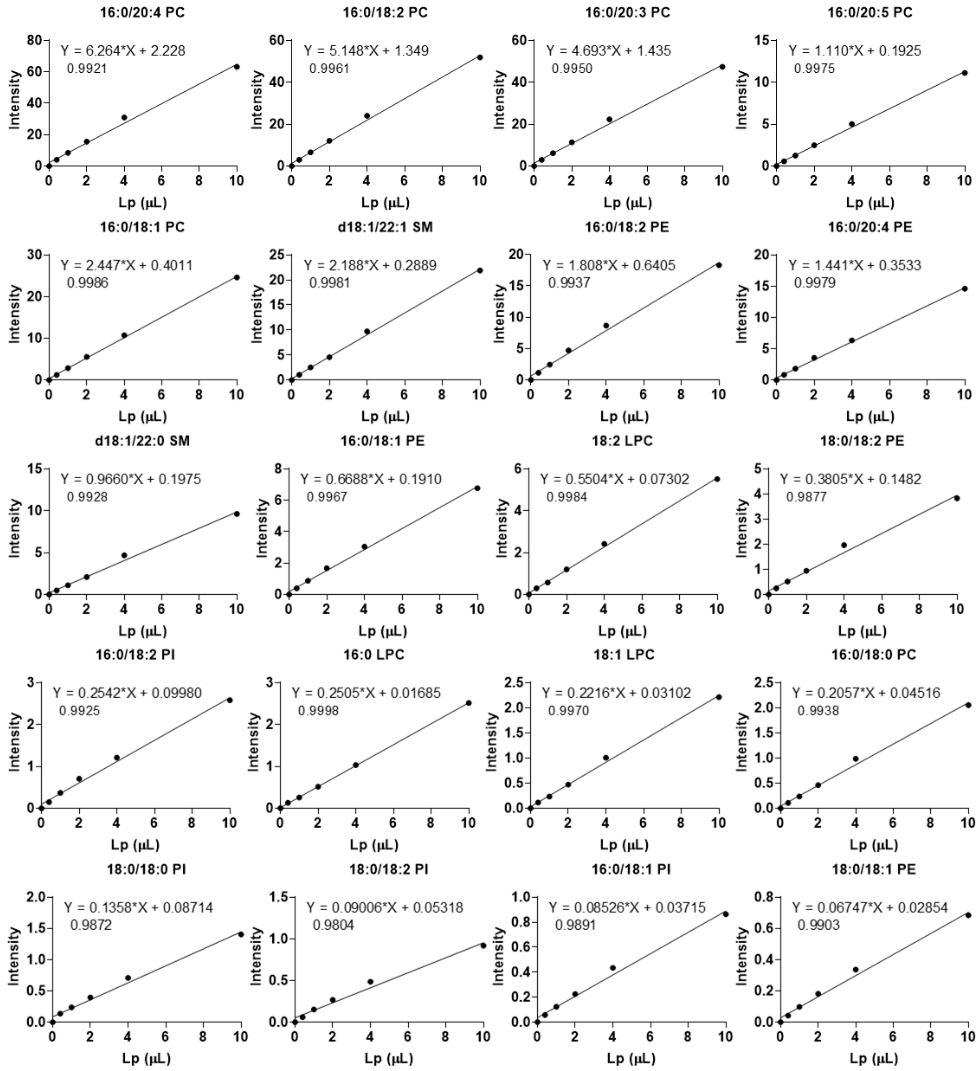

Figure S11. Correlation of the signal intensity of 20 major phospholipids and Lp content.

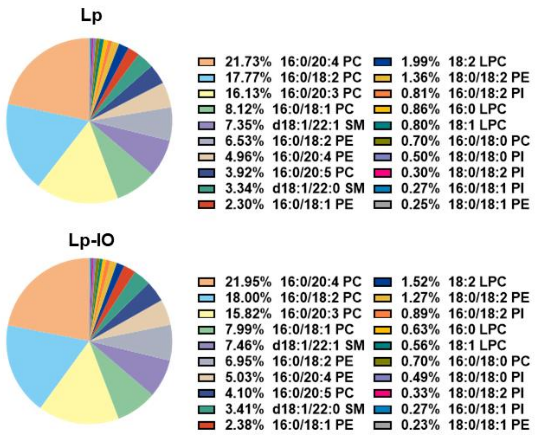

Figure S12. The peak area ratios for the major phospholipids in Lp and Lp-IO.

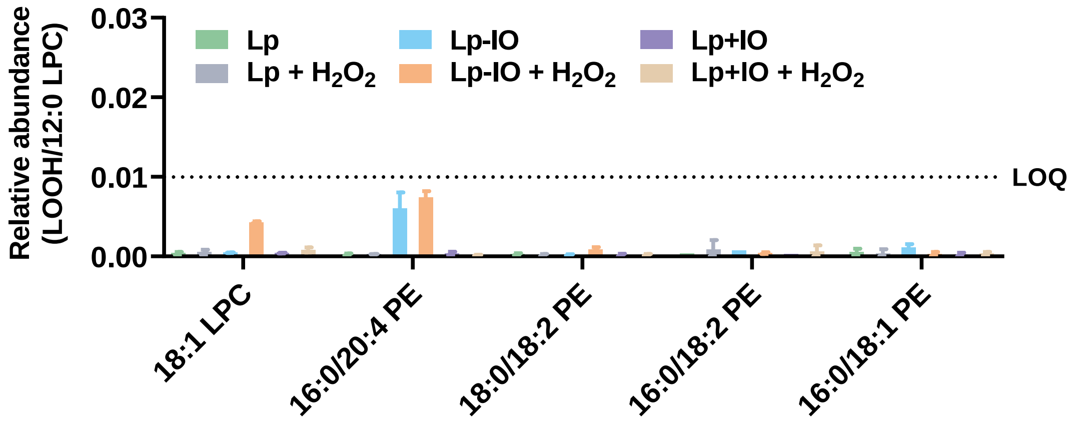

Figure S13. The peak area ratios of lipid peroxides (LOOH) to12:0 LPC (internal standard) for 18:1 LPC, 16:0/20:4 PE, 18:0/18:2 PE, 16:0/18:2 PE, and 16:0/18:1 PE in Lp, Lp-IO, and Lp+IO in the presence or absence of 1 mM H_2O_2 for 6 h, respectively. LOQ, the limit of quantification.

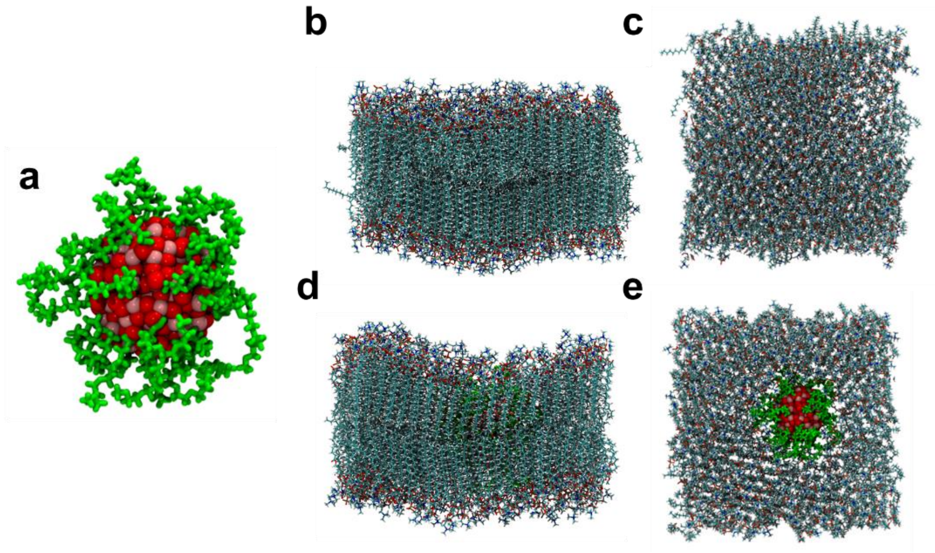

Figure S14. Profiles of 2 nm IO NPs decorated with 30 PEG chains (a), pure lipid bilayer (b, front view; c, top view), and IO-PEG dopped lipid bilayer (d, front view; e, top view).

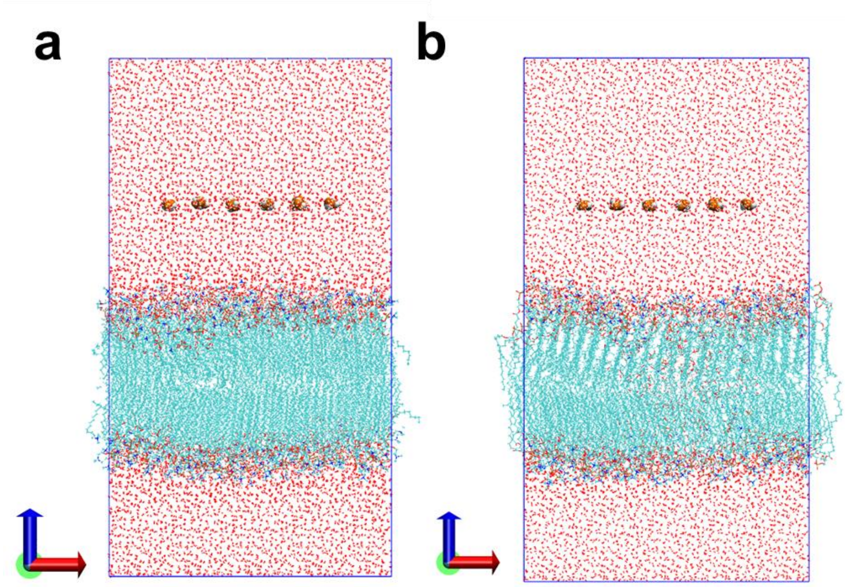

Figure S15. Simulation systems of H_2O_2 interacting with (a) pure lipid bilayer and (b) IO-PEG dopped lipid bilayer.

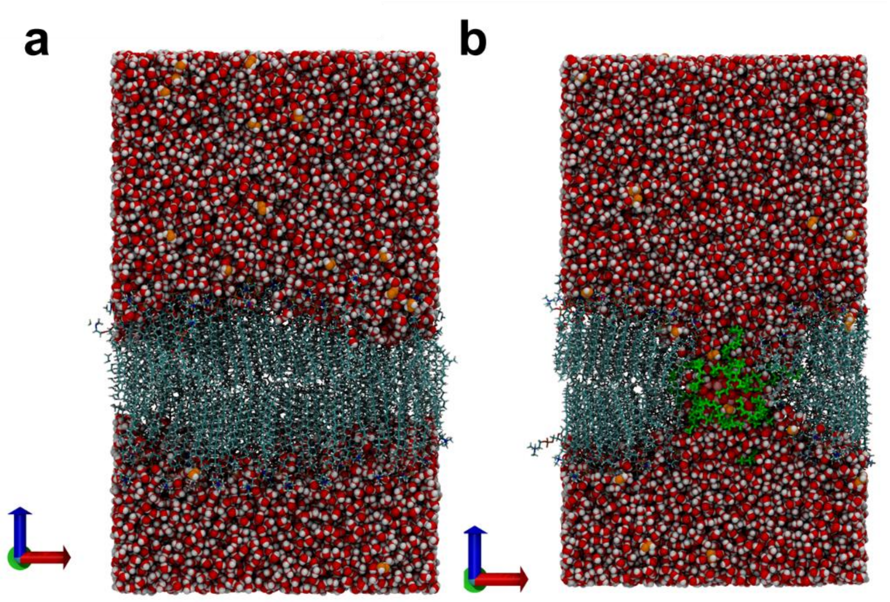

Figure S16. Typical equilibrated snapshots of H_2O_2 interacting with the pure lipid bilayer and IO-PEG dopped lipid bilayer.

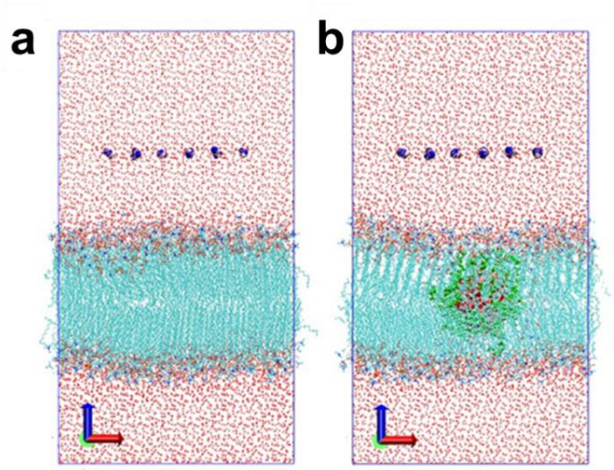

Figure S17. Simulation systems of •OH interacting with (a) pure lipid bilayer and (b) IO-PEG dopped lipid bilayer.

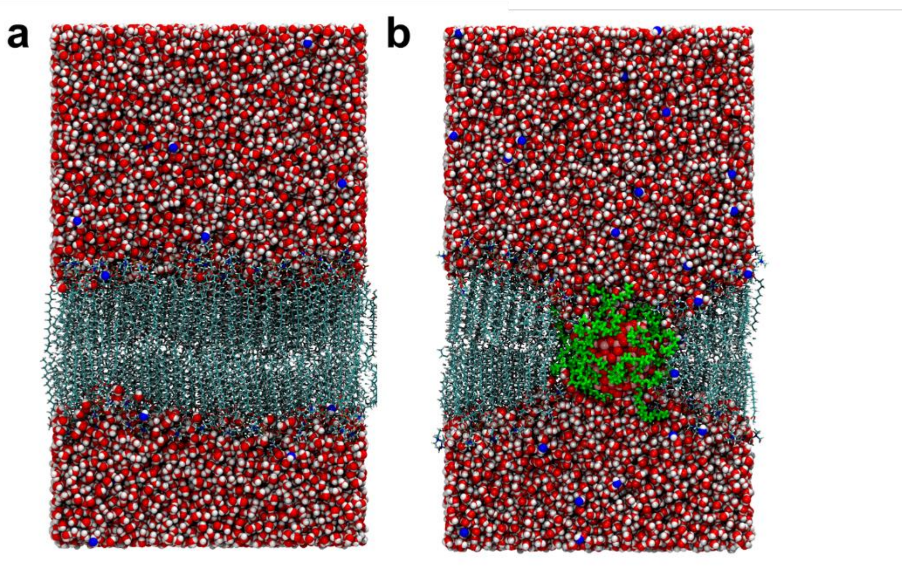

Figure S18. Typical equilibrated snapshots of •OH interacting with the pure lipid bilayer and IO-PEG dopped lipid bilayer.

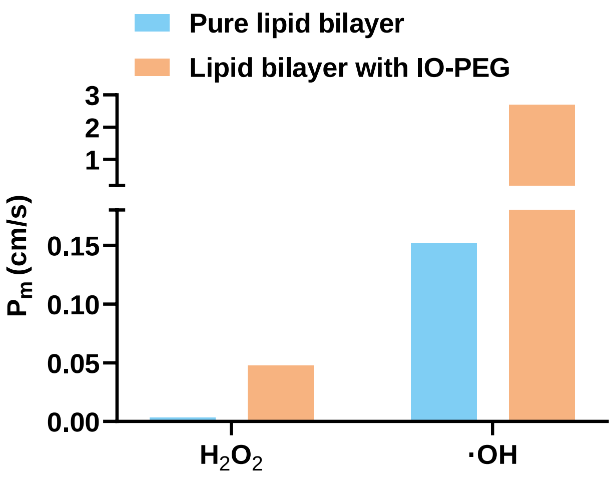

Figure S19. The calculated permeability coefficients (P_m) for H_2O_2 and •OH over the pure lipid bilayer and the IO-PEG dopped lipid bilayer, respectively.

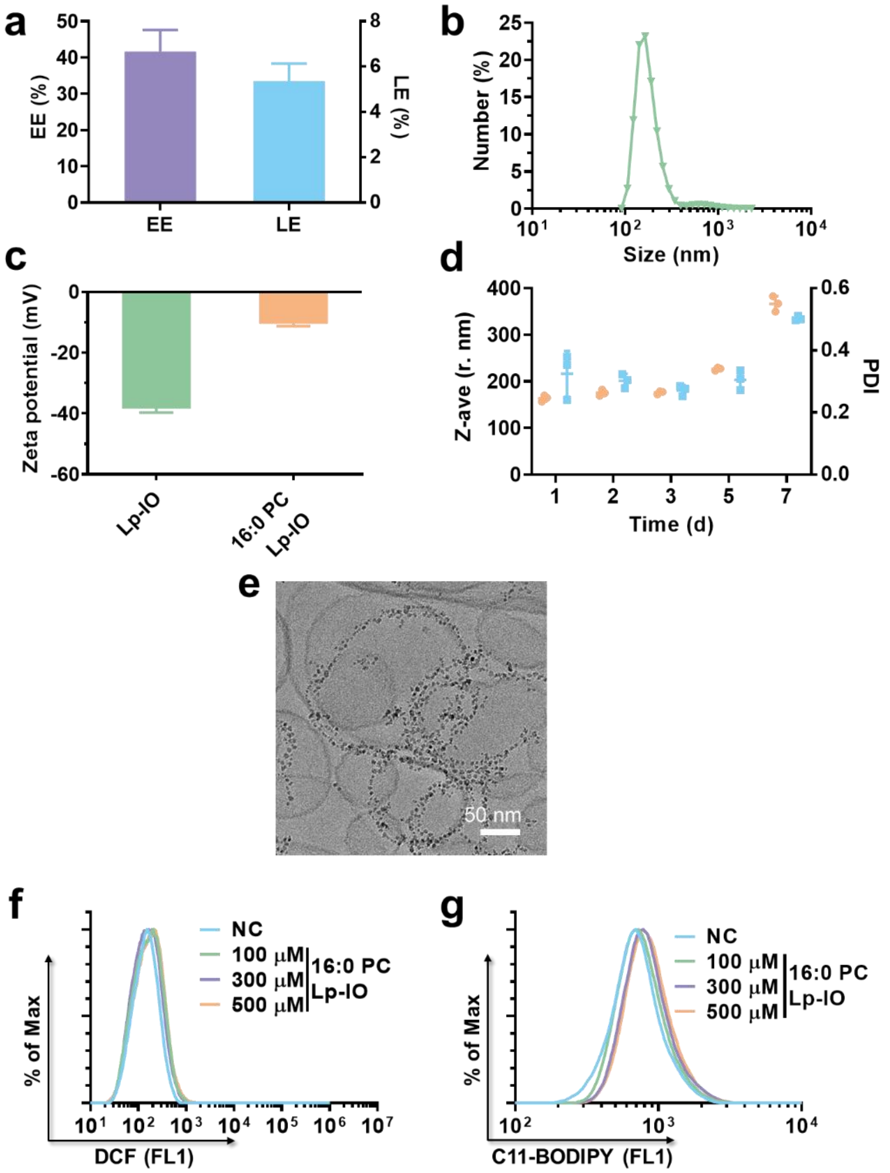

Figure S20. (a) Encapsulation efficiency and loading efficiency of 16:0 PC Lp for IO-PEG. (b) Hydrodynamic size distribution and (c) zeta potential of16:0 PC Lp-IO. (d) The stability of 16:0 PC Lp-IO in water at 37 ℃. (e) Cyro-TEM image of Lp-IO; the scale bar is 50 nm. Flow cytometry analysis of (f) ROSs and (g) LPOs in 4T1 cells treated with 16:0 PC Lp-IO (100, 300, 500 μM Fe) for 6 h, respectively.

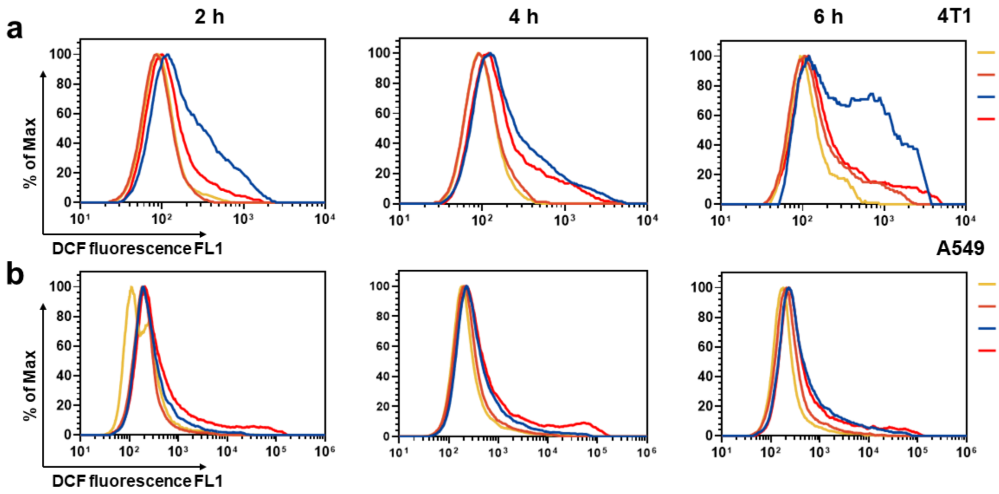

Figure S21. Flow cytometry analysis of ROSs in (a) 4T1 and (b) A549 cells treated with Lp (300 μg/mL), IO-PEG (300 μM Fe), Lp-IO (300 μM Fe), and RSL3 (5 μM) for 2, 4, 6 h.

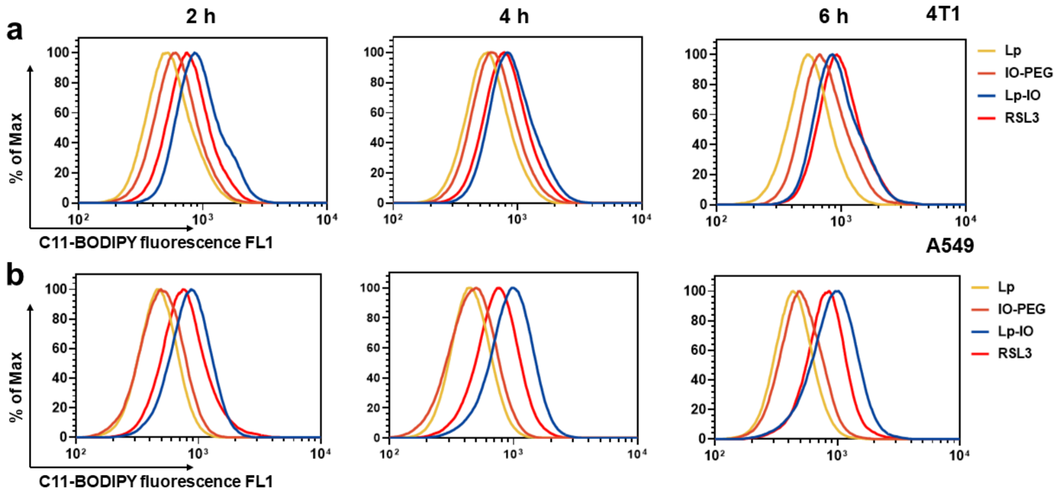

Figure S22. Flow cytometry analysis of LPOs in 4T1 (a) and A549 (b) cells treated for 2, 4, 6 h with Lp (300 μg/mL), IO-PEG (300 μM Fe), Lp-IO (300 μM Fe), and RSL3 (5 μM).

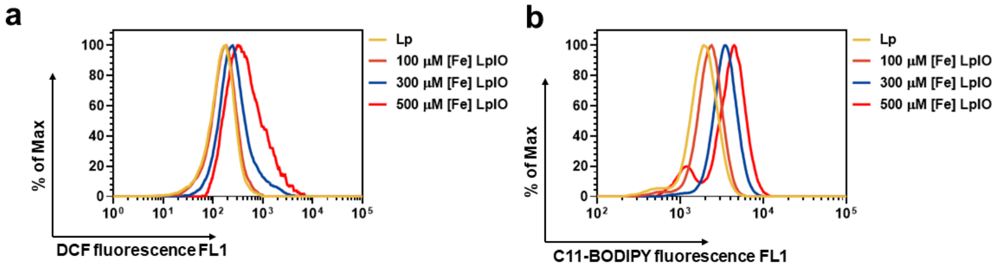

Figure S23. Flow cytometry analysis of (a) ROSs and (b) LPOs in A549 cells treated for 6 h with Lp (300 µg/mL) and Lp-IO (100, 300, 500 µM Fe), respectively.

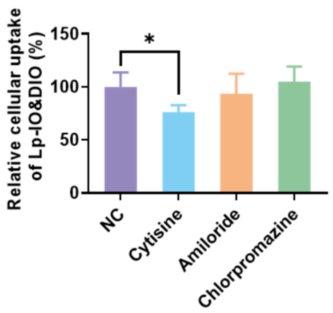

Figure S24. Relative cellular uptake of Lp-IO&Dio for 6 h in 4T1 cells treated with cytisine (inhibitor of caveolin-mediated endocytosis, 40 μg/mL), amiloride (inhibitor of macropinocytosis, 0.4 μg/mL), and chlorpromazine (inhibitor of grid-mediated endocytosis, 0.4 μg/mL), respectively.

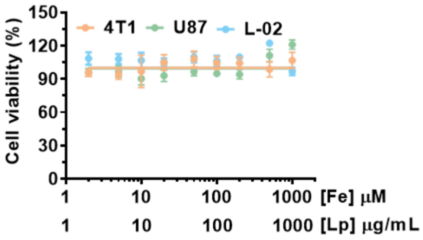

Figure S25. Viability of 4T1, U87, and L-02 cells separately treated with Lp+IO at different concentrations.

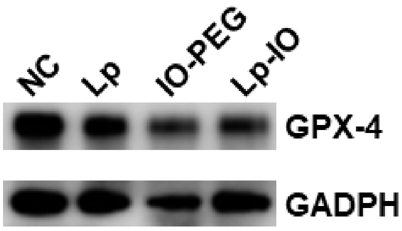

Figure S26. Western blot analysis of GPX-4 expression in 4T1 cells treated with Lp, IO-PEG, and Lp-IO for 6 h.

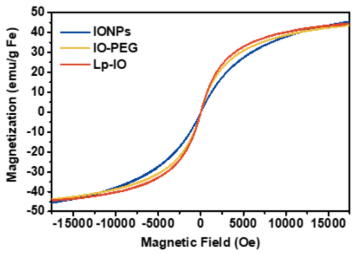

Figure S27. Magnetic hysteresis loops of IONPs, IO-PEG, and Lp-IO, respectively.

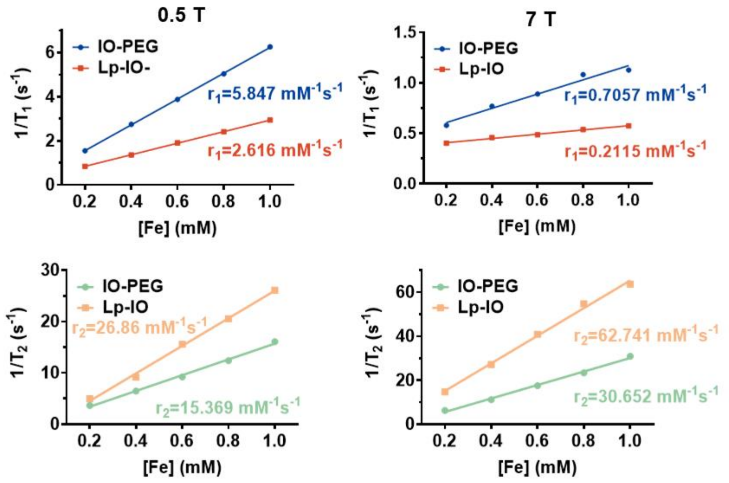

Figure S28. The plot of $1/T_1$ and $1/T_2$ over different iron concentrations of the IO-PEG and Lp-IO at 0.5T and 7.0 T. The slopes of the fitting line represent longitudinal relaxivity (r_1) and transverse relaxivity (r_2), respectively.

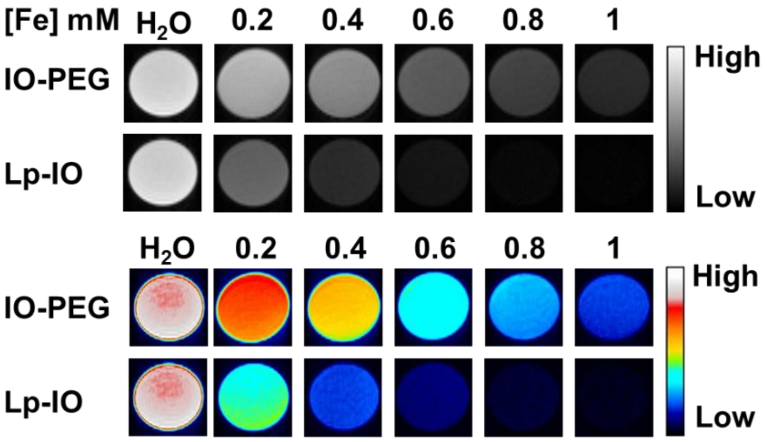

Figure S29. T_2-weighted MR images (top) and color contrasted image (bottom) of IO-PEG and Lp-IO at gradient concentrations. Pure H_2O was utilized as the control group.

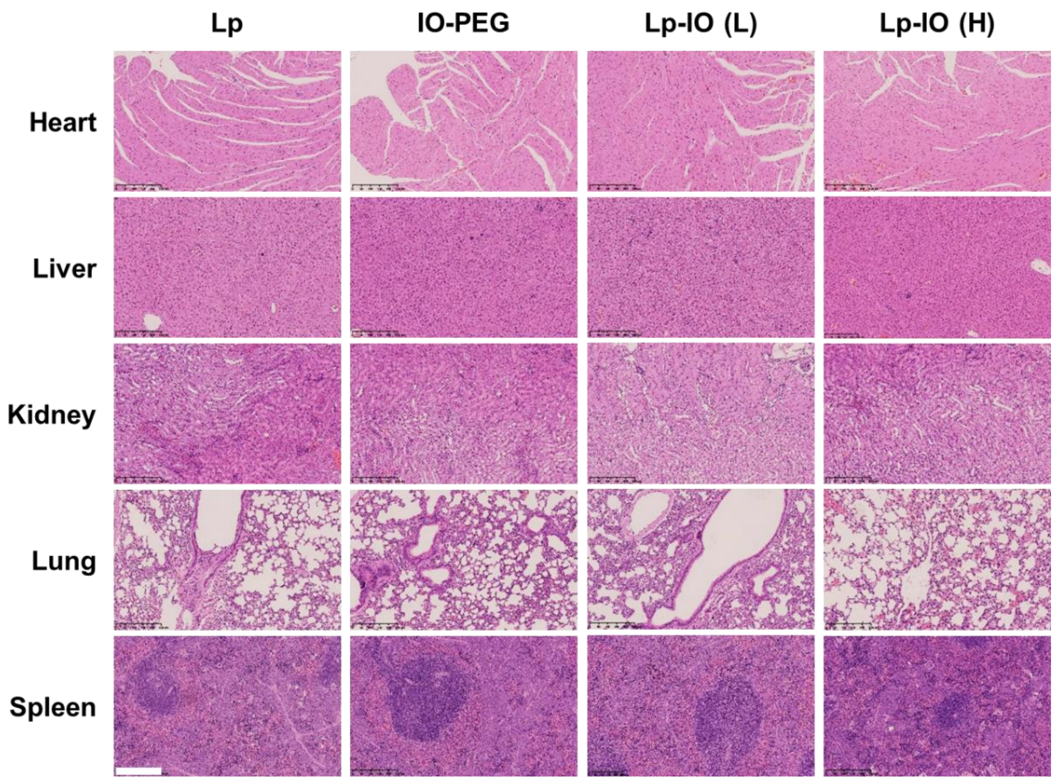

Figure S30. The H&E stained slices of heart, liver, kidney, lung, and spleen tissues from the mice on the 19th day. The scale bar is 250 μm.

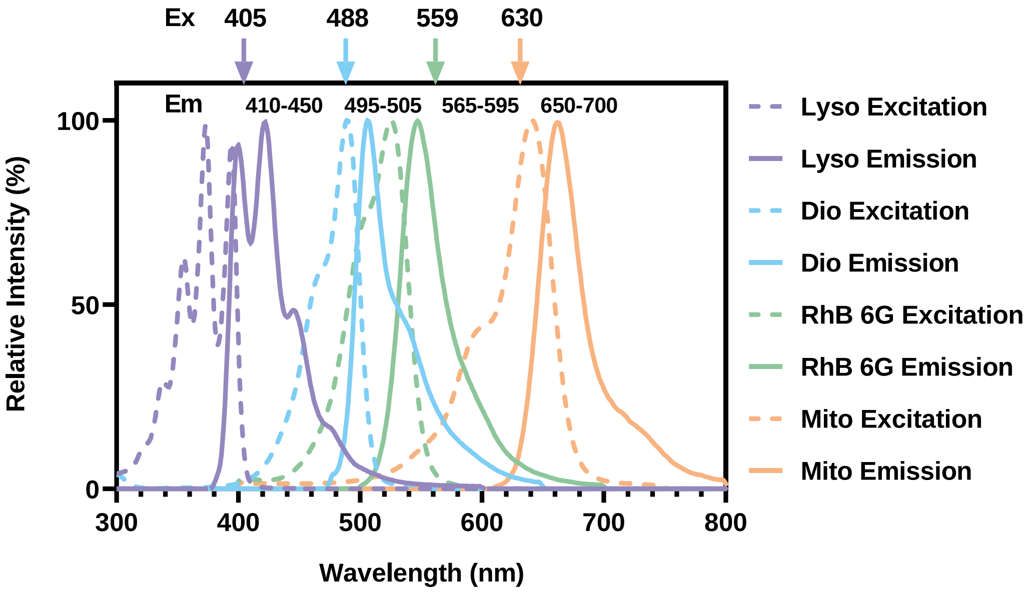

Figure S31. Excitation spectra, emission spectra, selected excitation wavelengths, and recorded emission wavelengths of LysoTracker™ Blue DND-22 (Lyso), biofilm dye-3,3'-Dioctadecyloxacarbocyanine Perchlorate (Dio), rhodamine 6G (RhB 6G), and MitoTracker™ Deep Red FM (Mito), respectively. The spectra data are derived from Fluorescence SpectraViewer (https://www.thermofisher.cn/order/fluorescence-spectraviewer#!/).

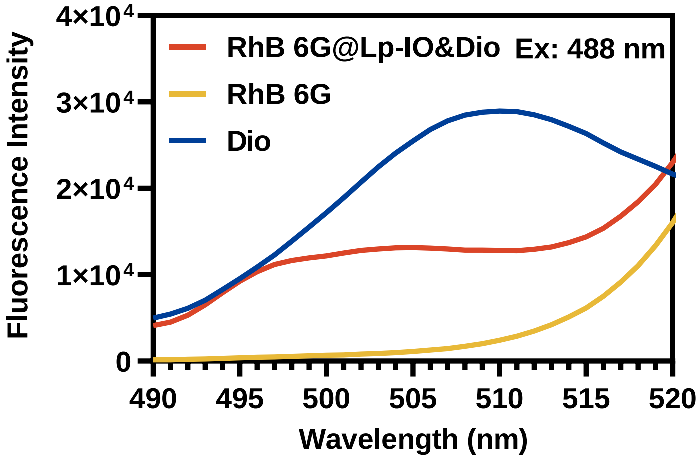

Figure S32. Emission spectra of RhB6G@Lp-IO&Dio with an excitation wavelength of 488 nm.

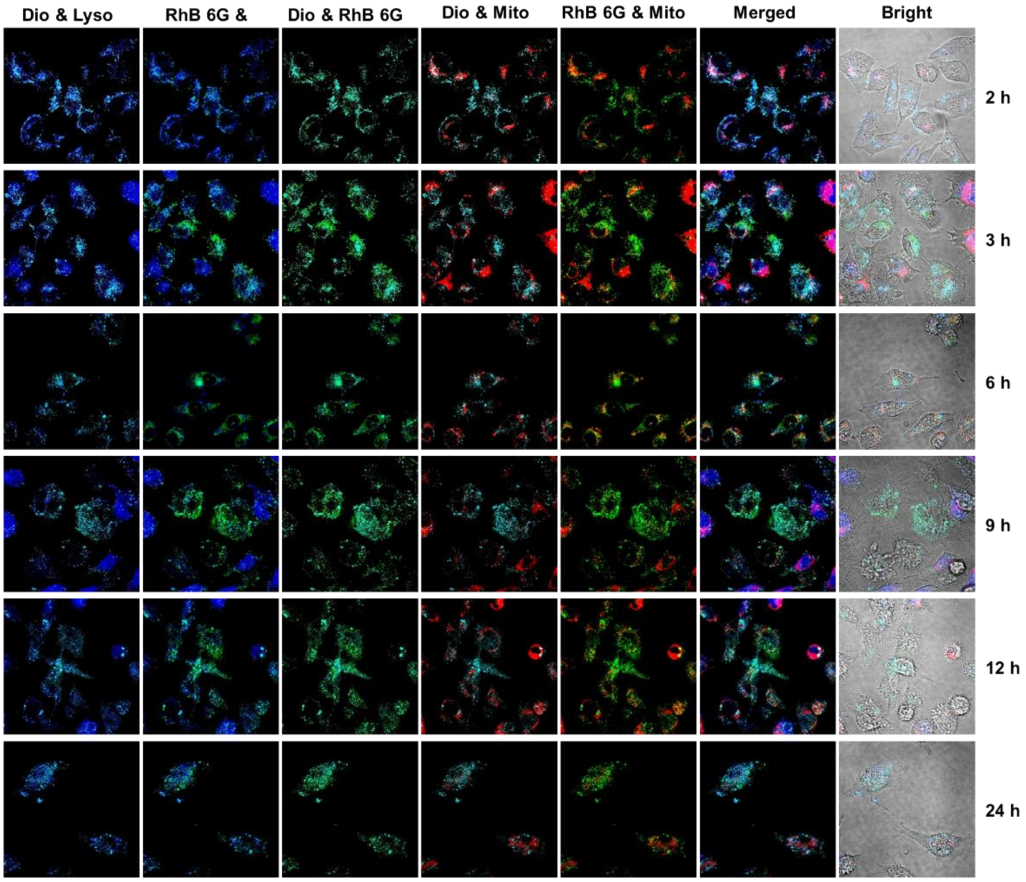

Figure S33. Merged FL images of 4T1 cells treated with RhB6G@Lp-IO&Dio for different intervals and subsequently stained with LysoTracker™ Blue and MitoTracker™ Deep Red.

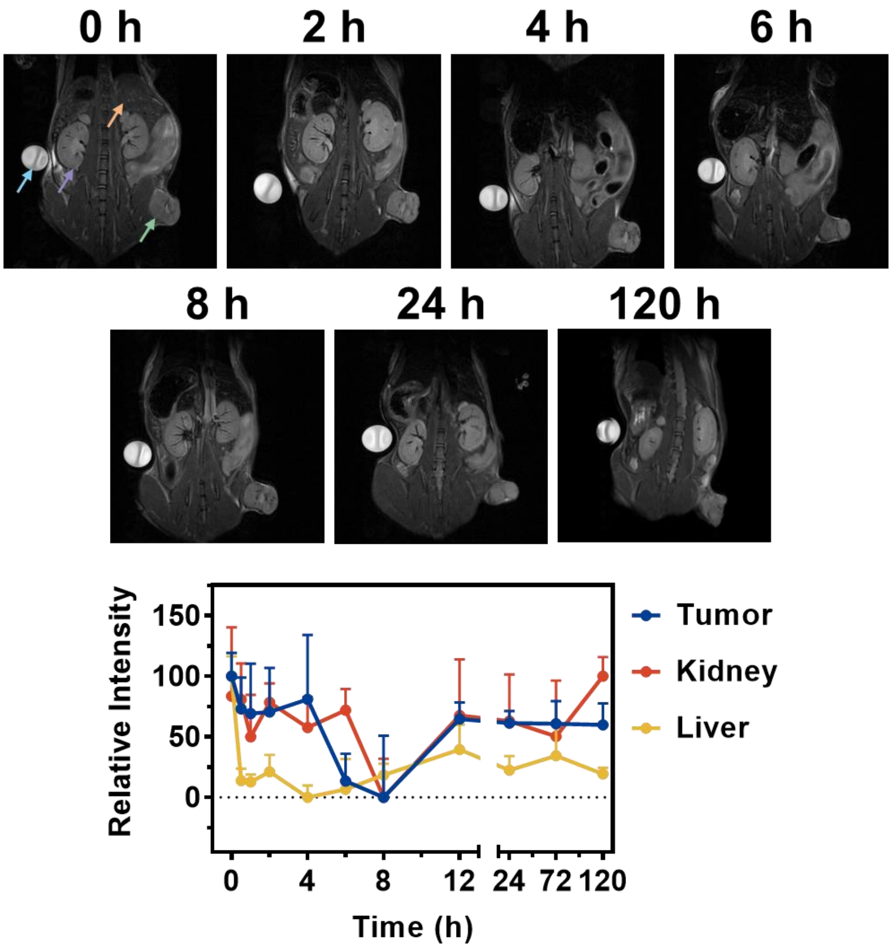

Figure S34. T_2-weighted MR images of 4T1 tumor-bearing Balb/c mice injected with DOX@Lp-IO at 2.5 mg Fe/kg for 0, 2, 4, 6, 8, 24, and 120 h. Light blue, lilac, light orange, and green arrows indicate water, kidney, liver, and tumor, respectively.

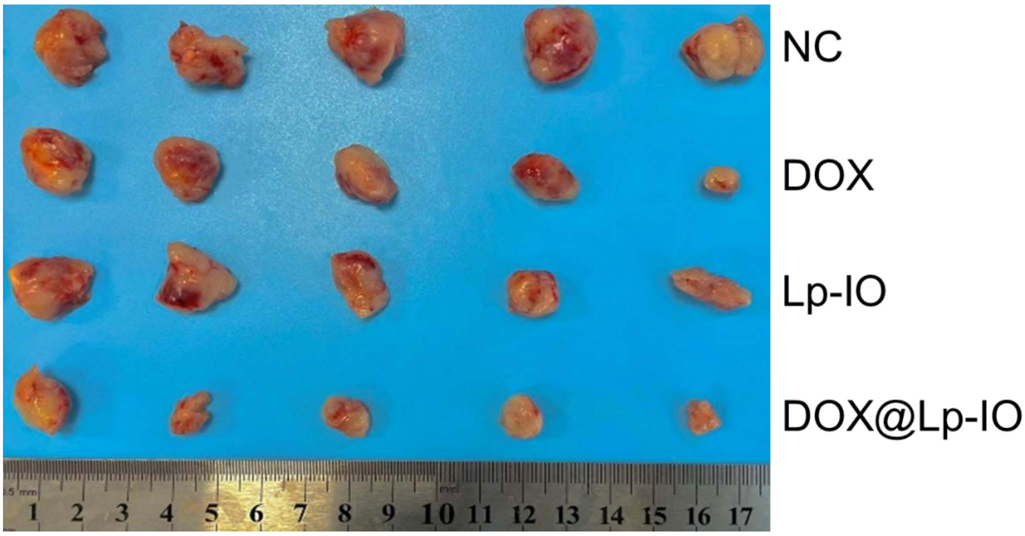

Figure S35. Photos of the dissected tumors from the mice treated with NC, DOX, Lp-IO, and DOX@Lp-IO on the 22nd day.

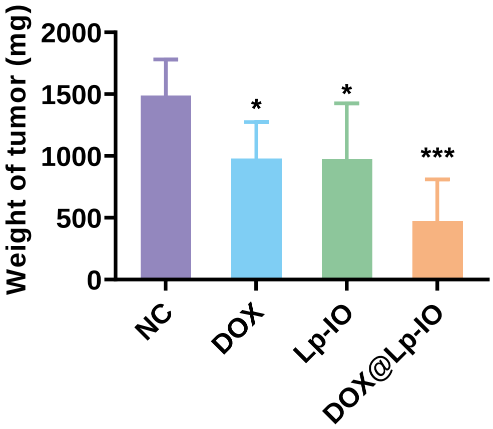

Figure S36. Tumor weight of the dissected tumors from the mice treated with NC, DOX, Lp-IO, and DOX@Lp-IO on the 22nd day.

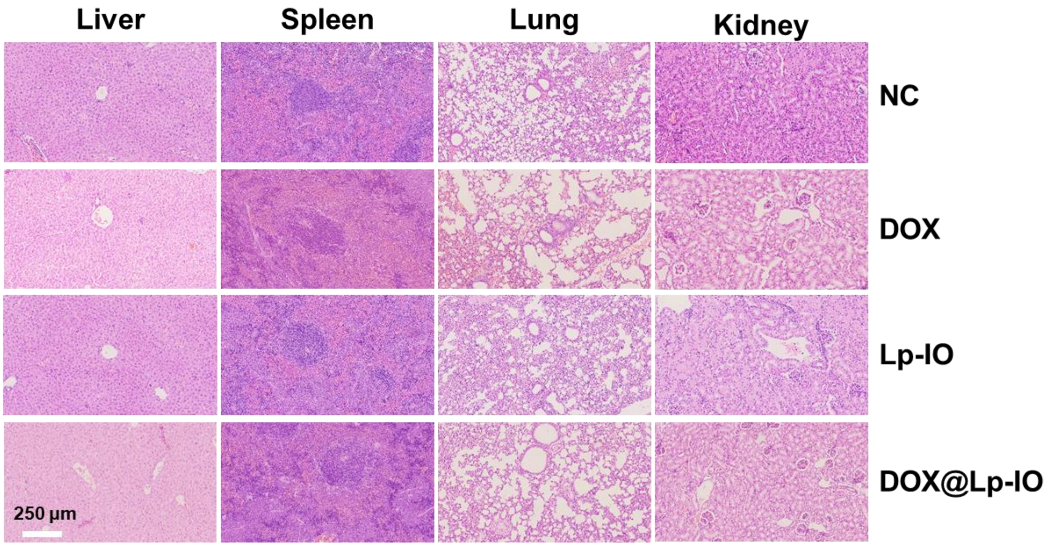

Figure S37. The H&E stained slices of major organs from the mice on the 19th day. The scale bar is 250 µm.

Supplementary Table

Table S1. Mass spectrometer parameters used for detection of lipids and lipid peroxide. BLOQ, below the limit of quantification.

Lipids and lipid peroxide	Precursor ion	Product ion	Retention time (min)
12:0 LPC<br>(1-lauroyl-2-hydroxy-sn-glycero-3-phosphocholine)	440.3	184	1.14
16:0/20:4 PC<br>(1-palmitoyl-2-arachidonoyl-sn-glycero-3-phosphocholine)	782.6	184	5.43
16:0/20:4 PC-OOH	814.6	184	3.24
16:0/20:3 PC<br>(1-palmitoyl-2-meadoyl-sn-glycero-3-phosphocholine)	784.6	184	6.5
16:0/20:3 PC-OOH	816.6	184	3.64
16:0/18:2 PC<br>(1-palmitoyl-2-linoleoyl-sn-glycero-3-phosphocholine)	758.6	184	6.46
16:0/18:2 PC-OOH	790.6	184	3.13
16:0/20:5 PC<br>(1-palmitoyl-2-eicosapentaenoyl-sn-glycero-3-phosphocholine)	780.6	184	4.67
16:0/20:5 PC-OOH	812.6	184	2.93
16:0/18:1 PC<br>(1-palmitoyl-2-oleoyl-sn-glycero-3-phosphocholine)	760.6	184	7.85
16:0/18:1 PC-OOH	792.6	184	3.62
18:2 LPC<br>(1-Linoleoyl-2-hydroxy-sn-glycero-3-phosphorylcholine)	520.3	184	1.47
18:2 LPC-OOH	552.3	184	1.04
18:1 LPC<br>(1-oleoyl-2-hydroxy-sn-glycero-3-phosphocholine)	522.3	184	1.89
18:1 LPC-OOH	554.3	184	BLOQ
16:0/20:4 PE<br>(1-palmitoyl-2-arachidonoyl-sn-glycero-3-phosphoethanolamine)	740.5	599.5	5.74
16:0/20:4 PE-OOH	772.5	631.5	BLOQ
16:0/18:2 PE<br>(1-palmitoyl-2-linoleoyl-sn-glycero-3-phosphoethanolamine)	716.5	575.5	6.89
16:0/18:2 PE-OOH	748.5	607.5	BLOQ
16:0/18:1 PE<br>(1-palmitoyl-2-oleoyl-sn-glycero-3-phosphoethanolamine)	718.5	577.5	8.4
16:0/18:1 PE-OOH	750.5	609.5	BLOQ
18:0/18:2 PE<br>(1-stearoyl-2-linoleoyl-sn-glycero-3-phosphoethanolamine)	744.5	603.5	6.92
18:0/18:2 PE-OOH	776.5	635.5	BLOQ
d18:1/22:1 SM<br>(N-docosenoyl-sphing-4-enine-1-phosphocholine)	785.6	184	6.49

d18:1/22:1 SM-OOH	817.6	184	3.64
d18:1/22:0 SM (N-docosanoyl-sphing-4-enine-1-phosphocholine)	787.6	184	6.5
d18:1/22:0 SM-OOH	819.6	184	4.25
16:0/18:2 PI (1-palmitoyl-2-linoleoyl-sn-glycero-3-phosphoinositol)	835.5	575.5	5.45
16:0/18:2 PI-OOH	867.5	607.5	BLOQ
16:0/18:1 PI (1-palmitoyl-2-oleoyl-sn-glycero-3-phosphoinositol)	837.5	577.5	5.45
16:0 LPC (1-palmitoyl-2-hydroxy-sn-glycero-3-phosphocholine)	496.3	184	1.82
16:0/18:0 PC (1-palmitoyl-2-stearoyl-sn-glycero-3-phosphocholine)	762.6	184	7.83
18:0/18:0 PI (1,2-distearoyl-sn-glycero-3-phosphoinositol)	867.5	607.5	15.3
18:0/18:1 PE (1-stearoyl-2-oleoyl-sn-glycero-3-phosphoethanolamine)	746.5	605.5	6.95

REFERENCES

1. Kim BH, Lee N and Kim H *et al.* Large-scale synthesis of uniform and extremely small-sized iron oxide nanoparticles for high-resolution T1 magnetic resonance imaging contrast agents. *J Am Chem Soc* 2011; **133**: 12624-31.

2. Li L, Wang L and Shangguan D *et al.* Ultra-high-performance liquid chromatography electrospray ionization tandem mass spectrometry for accurate analysis of glycerophospholipids and sphingolipids in drug resistance tumor cells. *J Chromatogr A* 2015; **1381**: 140-8.

3. Wand CR, Gibbon S and Siperstein FR. Adsorption of epoxy oligomers on iron oxide surfaces: the importance of surface treatment and the role of entropy. *Langmuir* 2021; **37**: 12409-18.

4. MacKerell AD, Bashford D and Bellott M *et al.* All-atom empirical potential for molecular modeling and dynamics studies of proteins. *J Phys Chem B* 1998; **102**: 3586-616.

5. Hess B, Kutzner C and van der Spoel D *et al.* GROMACS 4: algorithms for highly efficient, load-balanced, and scalable molecular simulation. *J Chem Theory*

Comput 2008; **4**: 435-47.

6. Humphrey W, Dalke A and Schulten K. VMD: visual molecular dynamics. *J Mol Graphics* 1996; **14**: 33-8.

7. Bemporad D, Essex JW and Luttmann C. Permeation of small molecules through a lipid bilayer: a computer simulation study. *J Phys Chem B* 2004; **108**: 4875-84.
